# The fate of notch-1 transcript is linked to cell cycle dynamics by activity of a natural antisense transcript

**DOI:** 10.1093/nar/gkab800

**Published:** 2021-09-14

**Authors:** Filip Vujovic, Saba Rezaei-Lotfi, Neil Hunter, Ramin M Farahani

**Affiliations:** IDR/Westmead Institute for Medical Research, NSW 2145, Australia; School of Medical Sciences, Faculty of Medicine and Health, University of Sydney, NSW 2006, Australia; IDR/Westmead Institute for Medical Research, NSW 2145, Australia; IDR/Westmead Institute for Medical Research, NSW 2145, Australia; IDR/Westmead Institute for Medical Research, NSW 2145, Australia; School of Medical Sciences, Faculty of Medicine and Health, University of Sydney, NSW 2006, Australia

## Abstract

A core imprint of metazoan life is that perturbations of cell cycle are offset by compensatory changes in successive cellular generations. This trait enhances robustness of multicellular growth and requires transmission of signaling cues within a cell lineage. Notably, the identity and mode of activity of transgenerational signals remain largely unknown. Here we report the discovery of a natural antisense transcript encoded in exon 25 of notch-1 locus (nAS25) by which mother cells control the fate of notch-1 transcript in daughter cells to buffer against perturbations of cell cycle. The antisense transcript is transcribed at G1 phase of cell cycle from a bi-directional E2F1-dependent promoter in the mother cell where the titer of nAS25 is calibrated to the length of G1. Transmission of the antisense transcript from mother to daughter cells stabilizes notch-1 sense transcript in G0 phase of daughter cells by masking it from RNA editing and resultant nonsense-mediated degradation. In consequence, nAS25-mediated amplification of notch-1 signaling reprograms G1 phase in daughter cells to compensate for the altered dynamics of the mother cell. The function of nAS25/notch-1 in integrating G1 phase history of the mother cell into that of daughter cells is compatible with the predicted activity of a molecular oscillator, slower than cyclins, that coordinates cell cycle within cell lineage.

## INTRODUCTION

Cell cycle is the basis not only for growth of metazoan animals but also for directing individual decisions (1) and collective behavior of proliferating cells (2) during developmental morphogenesis (3). This behavior predisposes to a metazoan ‘social dilemma’ as the cell-intrinsic (i.e. DNA replication) and the cell-extrinsic (i.e. self-organisation (2)) functions of cell cycle may occasionally clash. For example, cell cycle arrest to repair DNA damage would introduce noise into self-organisation dynamics unless compensatory mechanisms exist to offset the impact of such noise. There is evidence that transient expansion of a particular phase of cell cycle cannot be corrected by compensatory changes in the subsequent phases of the same cycle (4). However, cycling daughter cells adapt to altered kinetics of the mother cell by compensatory changes in cycle duration (5–7). Such compensation has to occur by communication of information regarding cycle dynamics of mother cell to daughter cells (8) and suggests involvement of transgenerational signals (5,6). Here we disclose that the post-transcriptional fate of Notch-1, a key regulator of cell cycle (9) in daughter cells is programmed by a natural antisense transcript that communicates G1 phase history of mother cells to daughter cells. In consequence, perturbations of G1 phase of mother cells are offset by Notch1-mediated compensatory changes in G1 phase of daughter cells.

Natural antisense transcripts (NATs) are endogenous transcripts that are characterized based on complementarity to other transcripts with known function (i.e. sense transcripts) (10). *Cis*-NATs exhibit perfect complementarity to sense transcripts that are transcribed from the same locus (11). Due to such complementarity, NATs could hybridize to sense transcripts with a high affinity. This could lead to diverse outcomes such as protection from degradation by masking or recruitment of RNA interference apparatus leading to degradation of sense transcripts (12). Apart from complementarity, bidirectional transcription of sense and antisense transcripts from the same locus sets a competition between these entities at transcriptional level, the outcome of which is to maintain robustness and to reduce stochasticity and intrinsic noise of gene expression (13). Here, we report that the *cis*-NAT of notch-1 determines the fate of notch-1 transcript by a combination of transcription-level and transcript-level interactions that are directly regulated via cell cycle dynamics, and in turn regulate the tempo of cell cycle in a notch1-dependent manner.

## MATERIALS AND METHODS

All chemicals were purchased from Sigma-Aldrich Inc. unless stated otherwise (Table 1). All primers, siRNAs and sgRNAs were purchased from IDT DNA.

**Table 1.** Reagents and resources used in this study

Reagent or resource	Source	Identifier
**Antibodies**		
Notch-1	Abcam	Cat# ab8925, RRID: AB_306863
Geminin	Abcam	Cat# ab195047, RRID: RRID:AB_2832993
Cyclin-D	Abcam	Cat# ab134175, RRID: AB_2750906
Ago-2	Abcam	Cat# ab156870, RRID: AB_2687492
Ki-67	Abcam	Cat# ab15580, RRID: AB_443209
E2F1	Abcam	Cat# ab4070, RRID: AB_304263
**Biological samples**		
Human brain pericytes	ScienCell	Cat# 1200, SUB9167017
**Chemicals**		
TMP	Sigma	Cat# T6137
Apcin	Sigma	Cat# SML1503
TAME Hydrochloride	Tocris	Cat# 4506
Roscovitine	Sigma	Cat# R7772
RO336	Sigma	Cat# SML0569
Crenigacestat	Selleckchem	Cat# S7169
**Software and algorithms**		
GraphPad Prism 7	GraphPad	https://www.graphpad.com/scientific-software/prism/
RStudio 1.3.959	RStudio	https://www.rstudio.com
**Deposited data**		
RNA-seq	This paper	GEO: GSE168092
Capture-seq	This paper	SRA: PRJNA705559

## EXPERIMENTAL MODEL AND SUBJECT DETAILS

### Cell culture

Human primary neural pericytes were purchased from ScienCell (Carlsbad, CA; #1200) and were cultured in Dulbecco's Modified Eagle Medium/F12 (DMEM/F12) supplemented with 10% fetal calf serum, recombinant human FGF-2 20 ng/ml (R&D Systems, 233-FB), and Antibiotic-Antimycotic (100×, Life Technologies).

## METHOD DETAILS

### Immunohistochemistry

Cells were fixed in 4% paraformaldehyde in 0.02 M phosphate buffer pH: 7.4 (680 mOsm), for 20 min at 4°C. After blocking in incubation buffer containing 0.1 M PBS, 1% BSA, 0.1% Tween-20 and 5% normal goat serum (for detection with rabbit Abs) or 5% normal rabbit serum (for detection with mouse Abs) for 40 min, sections were incubated with the primary antibodies overnight at 4°C and secondary antibodies for 1 h at room temperature. Specificity controls were carried out by incubating sections with rabbit or mouse IgG negative control antibodies.

### Gene expression analysis

RNA was isolated using RNeasy Mini Kit (Qiagen). Reverse transcription of the extracted RNA was carried out using SuperScript-III reverse transcriptase (200 units). Real-time PCR (38 cycles) was performed using SensiFAST™ SYBR^®^ Lo-ROX reagents (BIOLINE^®^) on a Stratagene^®^ Mx3000P real-time PCR instrument. Primers are listed in Supplementary Table S2.

### Programming of chromatin higher-order topology

To stabilise the higher-order topology of chromatin, cells were pre-incubated with 4,5ʹ,8-trimethylpsoralen (TMP, Sigma, final concentration: 20 μg/ml) in growth medium for 10 min in order to allow intercalation of TMP into negatively-supercoiled (underwound) chromatin domains (14,15). Cells were then placed on an ice bed and exposed to ≈3 kJ/m^2^ of 365 nm light for 30 s (ultra-high intensity UV-A lamp, Maxima: model ML-3500S) held at a distance of 20 cm as measured from the surface of the light filter to the surface of culture dishes. TMP-containing medium was replaced with fresh growth medium after 10 min and experiments were carried out as per the text.

### Quantification of locus topology

**Figure 1.**
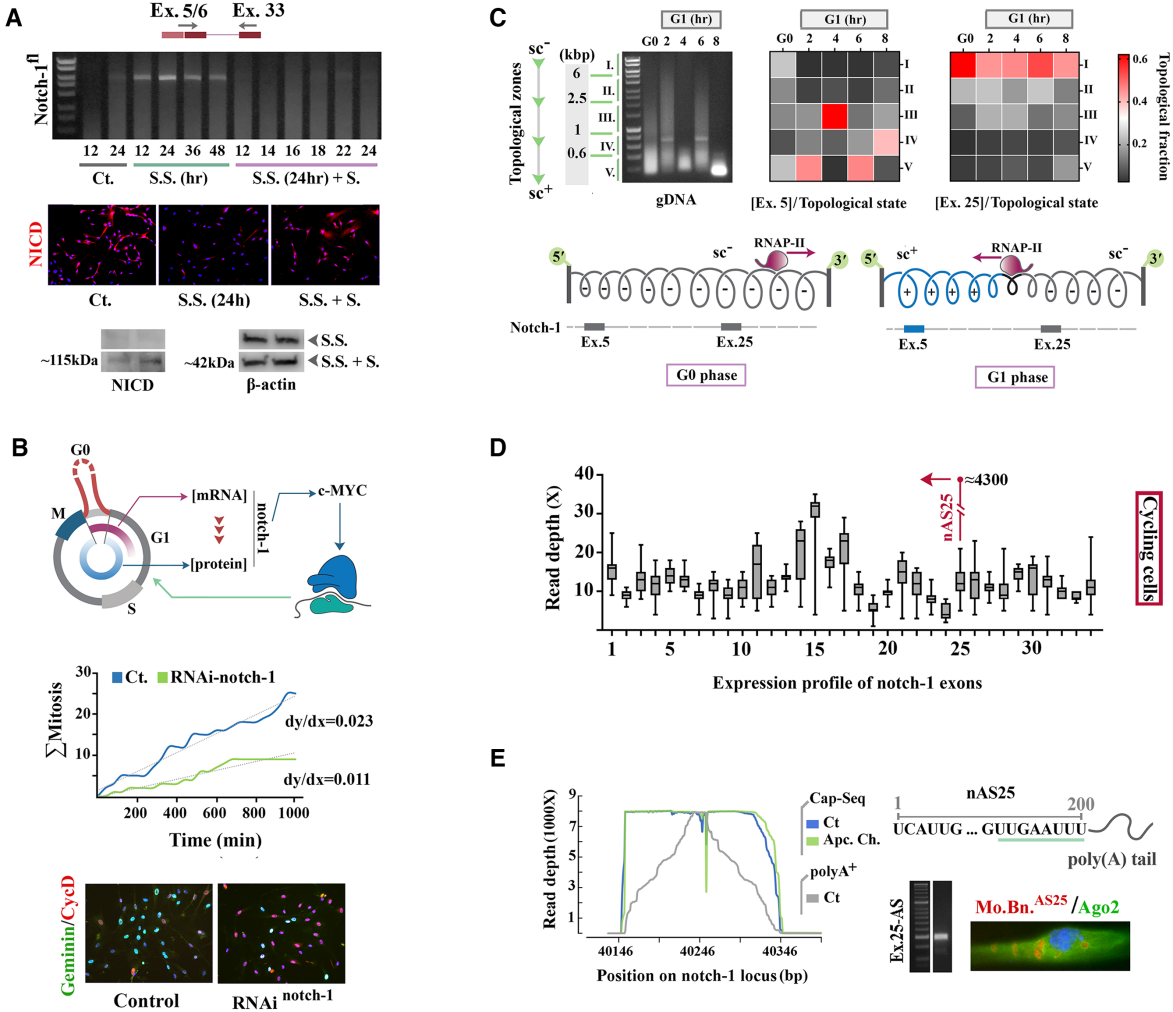
Transcriptional landscape of notch-1 locus is linked to progression of cell cycle. (**A**) Top gel shows PCR amplification of a notch-1 amplicon, spanning exons 5–33, in control cells, after synchronisation by serum starvation (S.S.) and after entry into cycle upon addition of serum (S.S.+S., unit: hour). Bottom micrograph shows detection by IHC of Notch-1 intracellular domain (NICD) in G0-synchronised (S.S.) and cycling (S.S.+S.) cells. The immunoblots show NICD and β-actin in G0-synchronised (S.S for 16 h and 24 h) and cycling cells (S.S. + S. (6 h, 12 h)). (**B**) Top schematic diagram shows the interplay of notch-1 and cell cycle dynamics whereby notch-1 is transcribed during G0. Upon progression to G1, RNA degradation and translation compete for a restricted pool of notch-1 transcripts generated at G0. By this mechanism, the level of pro-anabolic c-Myc and hence the tempo of G1 phase is precisely regulated. Middle graph shows cumulative mitotic rate of control and RNAi-notch-1 cells generated from single cell tracking (n = 150 cells). Slope of the fitted least-squares regression line (dotted lines) provides the linear approximation of the mitotic rate. Reduction of the average linear mitotic rate of RNAi-notch-1 cells and negative deviation from linearity of mitotic rate (2,36) after *t* = 700 min are consistent with decelerated cell cycle underpinned by prolonged G1. Cyclin-D1^high^/Geminin^low^ profile of cells upon RNAi-mediated suppression of notch-1 suggested transient arrest at G1. (**C**) Agarose gel shows remodeling of higher-order chromatin topology of synchronised cells upon entry to G1 phase (unit: hour). Heatmaps show normalised distibution of exons 5 and 25 in various topological states (zones I-V as per text). (**D**) Box plots show RNA-seq read depth for bases that map to notch-1 coding sequences (mean ± SD). Read depth of a short sequence (≈200 bp) within exon 25 was remarkably higher than the rest of exon 25. (**E**) Overlapping RNA-seq peak within exon 25 and the Capture-seq profiles (Ct. and Apcin-chase) of the antisense transcript show the precise position of poly-A^+^ nAS25 with a distinct terminal polyadenylation signal (green line). PCR amplification of nAS25 revealed a specific band at 200 bp and application of a molecular beacon specific to nAS25 (Mo.Bn.^AS25^) disclosed the cytoplasmic localisation of nAS25 in foci that were shown to be devoid of the RISC complex component Ago-2.

To study the higher-order topology of loci of interest, synchronised cultured cells were lysed at defined intervals. Subsequent to cell lysis, 4,5ʹ,8-trimethylpsoralen was employed to intercalate into negatively-supercoiled (underwound) DNA (14,15). The dishes containing the lysate were then placed on an ice bed and exposed to ≈3 kJ/m^2^ of 365 nm light for 40 s (ultra-high intensity UV-A lamp, Maxima: model ML-3500S) held at a distance of 15 cm, as measured from the surface of the light filter to the surface of cell lysate-containing dish. The extraction of DNA was then completed using a DNA isolation kit (ISOLATE II Genomic DNA Kit, Meridian Biosciences) according to the manufacturer's instructions. The isolated DNA was digested with MNase (2 U per million cells, NEB) for 5 min at room temperature. The enzyme was inactivated by adding EGTA. Final reaction products were analysed by DNA gel electrophoresis (1%) in the presence of chloroquine (5 μg/ml, Sigma) to distinguish differences in negative supercoiling densities and in control native gels. Five genomic fractions corresponding to the zones I-V of Figure 1C (refer to the main text) were then cut and purified from the gel using the Wizard SV Gel and PCR Clean-Up System (Promega). Isolated fractions were then incubated in a denaturing solution (0.5 M NaOH, 1.5 M NaCl) at 65°C for 60 min to reverse psoralen crosslinking. Specific PCR primers (as per Supplementary Table S2) were designed to quantify the distribution of the loci of interest (LoI) in four isolated genomic fractions. Real-time PCR (38 cycles) was performed using SensiFAST™ SYBR^®^ Lo-ROX reagents (BIOLINE^®^). Reaction mix comprised of 2 μl of gDNA, 400 nM primers, 10 μl of 2× SensiFAST SYBR Lo-ROX Mix, and 5 μl of PCR-grade water on a Stratagene^®^ Mx3000P real-time PCR instrument. Normalised representations of topological states of the LoI (Figure 1C of the main text) in zones I-V (Z_i_, i: 1-5) were generated based on the following formula:

$$\begin{equation*}{\boldsymbol{LoI}}\_{\boldsymbol{\ }}{{\boldsymbol{Z}}_{\boldsymbol{i}}} = \frac{{{2^{\Delta {\boldsymbol{Ct}}\left( {{{\boldsymbol{Z}}_{\boldsymbol{i}}} - {{\boldsymbol{Z}}_1}} \right)}}}}{{\mathop \sum \nolimits_{{\boldsymbol{i}} = 1}^5 {2^{\Delta {\boldsymbol{Ct}}\left( {{{\boldsymbol{Z}}_{\boldsymbol{i}}} - {{\boldsymbol{Z}}_1}} \right)}}}}{\boldsymbol{\ }}\end{equation*}$$

where LoI_*Z*_*i*_ (*i*: 1–5) represent the normalised distribution of a locus of interest in zones I–V corresponding to various higher-order topological states that form a gradient from sc^–^-dominated zone I, with low electrophoretic mobility, to sc+-dominated zone V, with high electrophoretic mobility.

### Small molecule inhibitors

The Cdk-2 inhibitor, Roscovitine (Seliciclib, Sigma R7772), was applied at a final concentration of 15 μM. The Cdk-1 inhibitor, RO-3306 (Sigma, SML0569), was applied at a final concentration of 2 μM. The selective Notch-1 inhibitor, Crenigacestat (LY3039478, Selleck chemicals), was applied at a final concentration of 10 μM.

### RNA interference

For small interfering RNA (siRNA)-mediated knockdown of nAS25, notch-1 (exons 34, 26 and 28), and Apobec-1, cells were electroporated with 200 nM of either the targeting or control siRNA. The sequence of siRNA targeting nAS25 is as follows:

sense: 5′-rGrGrArUrGrUrGrGrCrArCrArArGrArGrCrCrCrGrUrUrGAA-3′
antisense: 5′-rUrUrCrArArCrGrGrGrCrUrCrUrUrGrUrGrCrCrArCrArUrCrCrUrG-3′
The sequence of siRNA targeting notch1-exon34 is as follows:
sense: 5′-rCrArCrArGrGrArGrCrGrCrArUrGrCrArUrCrArCrGrArCAT-3′
antisense: 5′-rArUrGrUrCrGrUrGrArUrGrCrArUrGrCrGrCrUrCrCrUrGrUrGrCrG-3′
The sequence of siRNA targeting notch1-exon26 is as follows:
sense: 5′-rUrGrCrUrGrCrArCrArCrCrArArCrGrUrGrGrUrCrUrUrCAA-3′
antisense: 5′-rUrUrGrArArGrArCrCrArCrGrUrUrGrGrUrGrUrGrCrArGrCrArCrG-3′
The sequence of siRNA targeting notch1-exon28 is as follows:
sense: 5′-rGrGrCrGrGrCrArGrCrArUrGrGrCrCrArGrCrUrCrUrGrGTT-3′
antisense: 5′-rArArCrCrArGrArGrCrUrGrGrCrCrArUrGrCrUrGrCrCrGrCrCrGrG-3′

The sequence of siRNA targeting Apobec1 duplex is as follows:

sense: 5′-rGrGrCrArCrArUrGrGrArUrCrArArCrArArArArUrCrGrGCA-3′
antisense: 5′-rUrGrCrCrGrArUrUrUrUrGrUrUrGrArUrCrCrArUrGrUrGrCrCrUrU-3′

For electroporation in RNAi, cells were harvested, mixed with Dsi-RNA (i.e. siRNA) and resuspended in 400 μl of electroporation buffer (10^6^ cells/400 μl). Electroporation buffer comprised 20 mM HEPES, 135 mM KCl, 2 mM MgCl_2_, 0.5% Ficoll 400 and 2 mM ATP/5 mM glutathione (pH 7.6). Electroporation was carried out at 1700 V/cm, 700 μs, four pulses at 1-s intervals.

### Molecular beacon

The molecular beacon (Mo.Bn.^AS25^) was designed as described elsewhere (16). Mo.Bn.^AS25^ is labeled with a 6-FAM reporter dye at the 5′ end and an Iowa Black FQ quencher at the 3′ end (Integrated DNA Technologies) and has the sequence:

mCmUmUmCmC*mA*mG*mA*mA*mC*mU*mG*mC*mG*mU*mG*mC*mA*mG*mC*mG*mC*mG*mU*mC*mA*mA*mU*mG*mA*mC*mGmAmAmG (m represents 2′-O-methyl RNA modification; * represents PS linkage modification).

### Capture-sequencing of nAS25

A 5′-biotinylated oligo was designed to hybridise to nAS25 based on the following sequence:

5′-CTCTTGTGCCACATCCTGGACTACAGCTTCGGGGGTGGGGCCGGGCGCGACATCCCCCCGCCGCTGATCGAGGAGGCGTGCGAGCTGCCCGAGTGCCAGGAGGACGCGGGCAACAAGGTCTGCAGCCTGCAGTGCAACAACCACGCGTGCGGCTGGGACGGCGGTGACTGCTCCCTCAACTTCAATGACCCCTG-3′

Streptavidin Magnetic Beads (NEB) were utilised to purify nAS25. Th purified nAS25 was then sequenced as described in the next section.

### RNA-Seq analysis

RNA sequencing was performed by Ramaciotti Centre for Genomics (UNSW Sydney, Australia). Total RNA was extracted using the RNeasy Mini Kit (Qiagen). RNA integrity was evaluated using the Agilent 2100 Bioanalyzer (Agilent). After enrichment of Poly-A^+^ mRNA, the libraries were constructed using the NEBNext Ultra II RNA Library Prep Kit. Then the libraries were sequenced on Novaseq 6000 sequencing platform (Illumina). To perform transcript-level expression analysis, RNA-seq reads were mapped to the *Homo sapiens* GRCh38 reference transcriptome using Hisat2. StringTie was utilized to assemble and quantify the expressed transcripts. The transcript abundance and differential expression tables were generated using Ballgown. Finally, the statistical analysis of between-group differences was performed using EdgeR package. Gene ontology (GO) enrichment analysis was done using the Metascape, NetworkAnalyst and the Reactome databases. After analysis, GO clusters with *P* < 0.01 were selected and the log_2_ (fold_change) for transcripts in the cluster were extracted from the EdgeR table and graphed as a scatter plot where the values are normalized to the control group.

### Chromatin immunoprecipitation

For ChIP, 5 × 10^6^ cells were fixed with ice-cold 1% formaldehyde for 10 min at RT and the cross-linking was quenched with 125 mM glycine for 5 min at RT. Sonication was performed using a Covaris S220 to create ≈200–300 bp fragments. Chromatin was then immunoprecipitated with anti-E2F1 antibody (Abcam, cat no. ab4070). Chromatin was then incubated overnight at 4°C with protein G-coated Sepharose beads (Sigma). Beads were then washed twice with each of the following buffers: low-salt, high-salt, and TE wash buffers. DNA was subsequently eluted and digested with proteinase K (20 μg/μl) for 2 h at 55°C and incubated overnight at 65°C to reverse cross-links. Pure DNA was isolated using the ISOLATE II Genomic DNA Kit and PCR was performed using Phusion^®^ High-Fidelity DNA Polymerase (NEB).

### Synthesis and application of exogenous nAS25

In order to synthesize nAS25, the genomic region corresponding to A^241^:A^440^ of exon 25 was amplified using Phusion^®^ High-Fidelity DNA Polymerase (NEB) and with inclusion of a T7 RNA polymerase promoter. The amplified product was purified, and the fidelity was confirmed using Sanger sequencing. We then utilised the RiboMAX™ Large Scale RNA Production System (Promega) to synthesize nAS25 using the purified product as a template according to the manufacturer's instructions. The synthesized RNA was purified and introduced into cultured cells using electroporation.

### Application of Crispr-Cas9 and Crispr-dCas9

Guiding dCas9 to block the E2F1 binding site was achieved by designing an sgRNA that was complementary to 5′-CGGCAACCCCTGCTACAACC-3′. Cas9-mediated cleavage of notch-1 locus to uncouple transcription of nAS25 from the upstream DNA was achieved by designing an sgRNA that was complementary to 5′-CCCGATCTTTCTCTGTTGGTA-3′. The sgRNA, Alt-R^®^ S.p. HiFi Cas9 Nuclease and Alt-R^®^ S.p. dCas9 protein were purchased from IDTDNA. SgRNA and the enzymes were resuspended in Nuclease-Free Duplex Buffer and Cas9 buffer (20 mM HEPES; 150 mM KCI, pH 7.5), respectively. Ribonucleoprotein (RNP) complex was prepared by combining the sgRNA (final concentration: 120 pmol) and Cas9/dCas9 enzymes (final concentration: 104 pmol) and incubation at RT for 20 min. For electroporation, cultured cells were harvested, mixed with RNP and resuspended in 400 μL of electroporation buffer (10^6^ cells/400 μl). Electroporation buffer comprised 20 mM HEPES, 135 mM KCl, 2 mM MgCl_2_, 0.5% Ficoll 400, and 2 mM ATP/5 mM glutathione (pH 7.6). Electroporation was carried out at 1700 V/cm, 700 μs, four pulses at 1-s intervals. After introduction of the sgRNA and Cas9/dCas9, cells were returned to growth medium and incubated for an additional 12h.

## QUANTIFICATION AND STATISTICAL ANALYSIS

SPSS statistical software (SPSS v.16, Chicago, IL, USA) was used for the statistical analysis of data. The relative expression levels of genes of interest were compared using univariate ANOVA and non-parametric Mann–Whitney *U* test. The results of statistical analysis can be found in figure legends. Plots were generated using Prism software. Data are presented as mean ± SD. In the present study, a *P*-value < 0.01 (*) was considered as statistically significant.

## RESULTS

### Cell cycle regulates the temporal transcriptional landscape of notch-1

Upon analysing the temporal expression profile of human notch-1 locus, we noted an anomaly. The full notch-1 transcript (ENST00000651671.1 encoding the functional Notch-1 protein that contains 36 EGF-repeat domains, and 6 Ankyrin repeat domains) was not readily detectable in cycling cells (Figure 1A). However, synchronisation of cells at G0 phase of cell cycle by serum deprivation disclosed the transcript. Once again, the transcript became undetectable as G0-synchronised cells progressed into interphase after addition of serum (Figure 1A). Notably, degradation of notch-1 transcript at interphase coincided with emergence of the translated protein (Figure 1A). We reasoned that restriction of transcription of notch-1 to G0 would facilitate titration of the protein at G1 by defining the pool of notch-1 transcript available to translation machinery. Precise titration of Notch-1 at G1 phase is essential given the pivotal role of this protein in inducing c-Myc (17), the master pro-anabolic driver of G1 phase (18). Corroborating this notion, RNAi-mediated depletion of notch-1 transcript decreased the average mitotic rate of cycling cells by decelerating G1 phase dynamics, as characterised by enrichment of Cyclin-D1^high^/Geminin^low^ cells (Figure 1B, Supplementary Figure S1). It was logical that notch-1 expression must be calibrated at G0 to regulate the tempo of G1 phase. However, it remained unclear how such calibration was achieved G0, a transitional phase that links the cell cycle of mother and daughter cells. One explanation was that M phase-specific remodelling of higher-order chromatin topology (19) renders notch-1 locus accessible and enables expression of full transcript at G0. To probe this possibility, we began by investigating the transcriptional landscape of notch-1 locus upon transition from G0 to G1.

It is known that the rotational torque generated by RNA polymerase-II (RNAP-II) during transcription elongation (20,21) induces positive supercoils in the DNA template ahead of it and negative supercoils behind it (21). We used this phenomenon to disclose RNAP-II polymerisation dynamics at notch-1 locus upon progression of cell cycle from M phase to G1. Synchronisation of cells was achieved by simultaneous application of the anaphase-promoting complex (APC) inhibitors (22), Apcin and tosyl-l-arginine methyl ester (TAME), for 16h and the subsequent release of M phase-synchronised cells into interphase propelled by washing out the inhibitors (termed Apcin-chase, Supplementary Figure S2). Higher-order chromatin topology of synchronised cells was captured by application of 4,5′,8-trimethylpsoralen (TMP)/UV-A(15), prior to extraction and fragmentation of DNA and qPCR. The distribution of notch-1 exon 5 and exon 25 was plotted in five genomic fractions corresponding to the zones I-V of Figure 1C. DNA in zone I (>6 kb) is hyper-negatively supercoiled characterised by low electrophoretic mobility with gradual transition to a positively supercoiled topology and high electrophoretic mobility in zone V (<0.6 kb). A key structural adaptation of notch-1 locus in transition from M phase to G1 phase was predominant adoption of a positively supercoiled topology by exon 5 at G1 phase (note the enrichment of zone V and depletion of zone I), relative to the topological distribution of the exon at G0 (two-tailed *P* < 0.0001, Figure 1C). In contrast, exon 25 retained negatively supercoiled configuration both at G0 and G1 (Figure 1C). This finding suggested that notch-1 locus becomes fully accessible at G0, potentially due to M phase specific remodelling of chromatin (19). The accessibility is revoked concurrent with the reversal of polymerisation activity of RNAP-II from sense to antisense direction upon progression of cell cycle to G1 phase (Figure 1C). To confirm this interpretation, we fingerprinted the transcriptional landscape of the notch-1 locus.

RNA-seq profile of poly(A)^+^ transcripts isolated from M phase-synchronised (Apcin/TAME-treated for 16h) and G0-synchronised cells (serum starvation, 24h, Supplementary Figure S3) revealed a short polyadenylated transcript (Figure 1D, Supplementary Figure S4) that corresponded to highly conserved A^241^: A^440^ of exon 25 (Figure 1D, Supplementary Figures S5, 6). Reverse transcription of the latter short transcript using an antisense-specific primer (see methods for details) followed by PCR amplification and Sanger sequencing revealed that the short transcript is a natural antisense transcript originating from exon 25 of notch-1 locus. We refer to this natural antisense transcript of exon 25 as **nAS25** throughout the manuscript. Capture-Seq using antisense-specific oligonucleotide bait and application of a molecular beacon designed to detect nAS25 provided further validation of the antisense identity of nAS25. A further corollary of transcribing nAS25 in the antisense direction was that the exonic profile downstream to exon 25 (Figure 1D) could belong to a noncoding truncated notch-1 transcript. Application of RNAi designed to target exon 26 and exon 28 confirmed the latter notion (supplementary Figure S7). Findings suggested that transcription of notch-1 as a full transcript is confined to G0, an activity that is superseded in transition to G1 by transcription of nAS25 and the truncated noncoding notch-1 from a putative bidirectional promoter at exon 25. Accordingly, interrupted transcription of notch-1 as a full transcript at G1 could potentially be attributed to the convergence of polymerisation by RNAP-II from template and non-template strands of DNA (23,24), a scenario that was confirmed by subsequent experiments. While restriction of transcription of notch-1 to G0 defines the available pool of transcript at G1, precise calibration will not be achieved unless the balance of competition between degradation and translation of the transcript is also regulated. Hence, we next focused on degradation of notch-1 transcript at G1.

### RNA editing at G0 phase allocates the degradable pool of notch-1 transcripts

**Figure 2.**
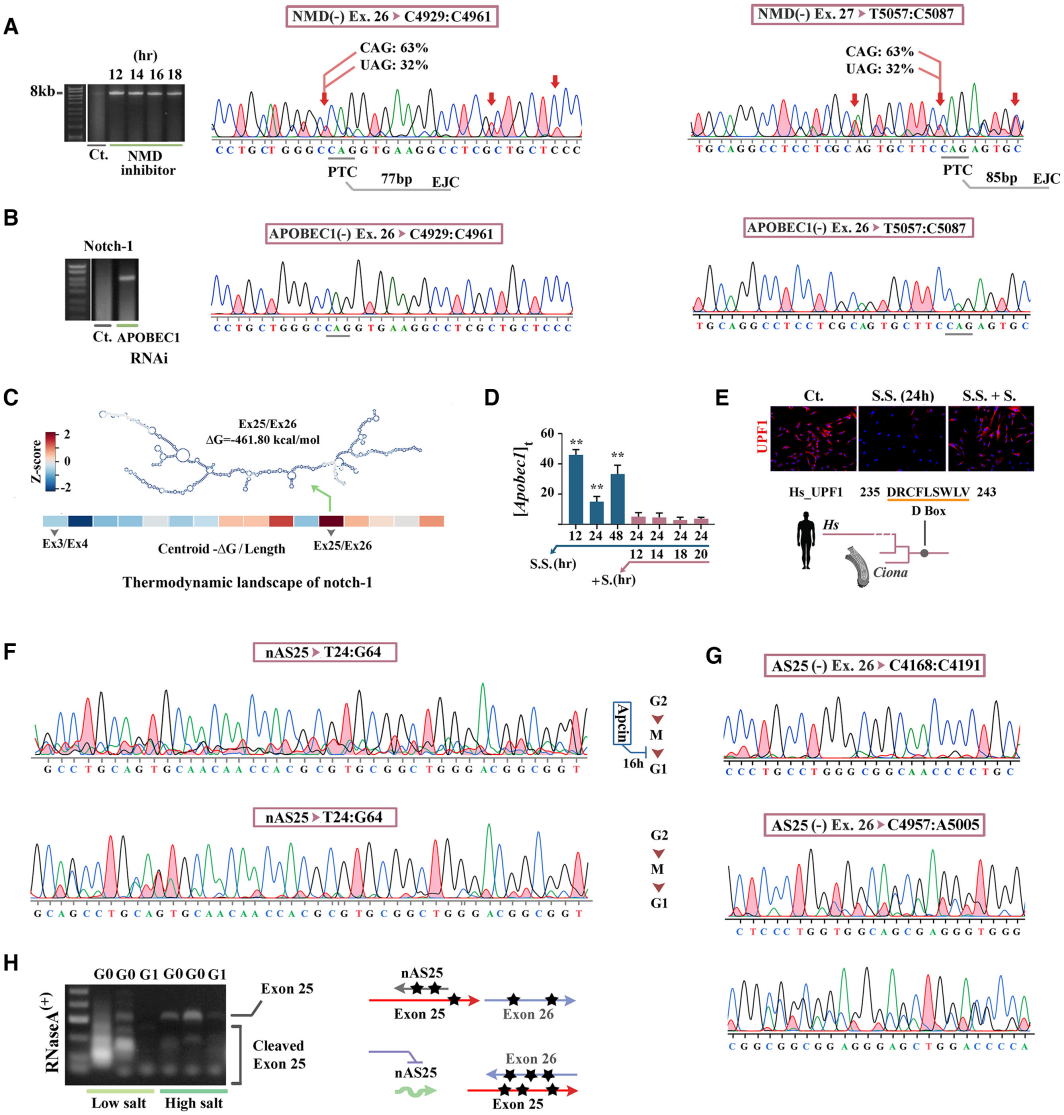
Epi-transcriptional regulation programs availability of notch-1. (**A**) Gel shows consistent detection of the notch-1 amplicon spanning exons 5–33 after inhibition of NMD (time points refer to the post-inhibition period). Two representative Sanger sequencing electropherograms show extensive base substitutions in exons 26 and 27 of notch-1 transcripts, recovered from NMD-inhibited cells. Premature stop codons (PTC) and their distance to downstream exon junction complex is also shown beneath the electropherograms. (**B**) Sanger sequencing electropherograms show absence of base substitutions in exons 26 and 27 of notch-1 transcript after RNAi-mediated inhibition of APOBEC1. (**C**) Heatmap shows propensity of notch-1 tandem exon pairs to form secondary structure. The thermodynamics propensities were estimated from minimum free energies of centroids formed by exon pairs, after normalisation to the combined length of exon pairs and final conversion to *z*-score. Note the high propensity of exon25/26 pair for secondary structure formation (top). (**D**) Expression level of Apobec1 in G0-synchronised (turquoise) cells and cycling cells (maroon) normalised to control cells. ** *P* < 0.001. (**E**) Micrograph shows detection by IHC of UPF-1 in G0-synchronised and cycling cells. Presence of a highly conserved (Supplementary Table S1) D-box sequence in UPF-1, recognised by APC/C, explains the absence of this protein in G0. (**F**) Sanger sequencing electropherograms of the region that corresponds to G26:G111 of nAS25 shows extensive editing of the antisense transcript in G0-synchronised cells and absence of extensive editing in nAS25 recovered from cycling cells. (**G**) Sanger sequencing electropherograms show extensive editing of exon 26 of notch-1 after RNAi-mediated depletion of nAS25. Note that the region upstream to the sequence encoding nAS25 (top electropherogram) remains relatively unaffected. (**H**) Gel shows PCR amplification of exon 25 of notch-1 in RNaseA-treated RNA samples isolated from G0-synchronised (serum starvation 16 h, 24 h) and G1 phase cells (following addition of serum). In G0-synchronised cells, nAS25 hybridises to exon 25 and protects it from catalytic activity of RNase-A at high salt (0.35 M NaCl) condition, as opposed to cleavage of exon 25 at low salt condition. Proposed model (right) for nAS25-mediated protection of exons 25–26 of notch-1 transcript via hybridization to exon 25 and disruption of secondary structure formation that facilitates RNA editing.

It is reported that nonsense-mediated decay (NMD) regulates availability of the major drivers of cell cycle, including c-Myc (25). Given the reported species of notch1 transcript that are degraded via NMD (Ensembl genome browser 104, transcripts related to ENSG00000148400), we asked if notch-1 is also regulated via NMD. After pharmacological inhibition of nonsense-mediated mRNA decay (NMD) by application of a validated specific SMG7 inhibitor (C_21_H_25_N_3_S: 100nM, 12–18h), the transcript encoding a major fraction of full notch-1 (exons 5–33, a proxy for notch-1) became readily detectable (Figure 2A). However, Sanger sequencing revealed that ≈32% of the transcripts recovered from NMD-inhibited cells were extensively edited in the GC-rich region (G∼C content: 68%) corresponding to exons 25–27 (Figure 2A). Deconvolution of the edited and non-edited notch-1 transcripts using Tracy package (26) disclosed two predominant cryptic premature termination codons (PTC) in exons 26 and 27, generated by C-to-U editing of cytosines C^4939^, C^5080^ (Figure 2A). The presence of PTCs explained NMD-mediated degradation of the edited notch-1 transcripts (27). RNAi-mediated inhibition of the C→U editing enzyme, APOBEC1, resulted in recovery of the non-edited full notch-1 transcript from cycling cells (Figure 2B). These findings suggested that APOBEC1 may regulate temporal availability of notch-1 mRNA by generating PTCs that trigger steady-state NMD-mediated degradation of the transcript during G1 phase. The conclusion was consistent with the observation that exons 25–27 of notch-1 transcript, aside from being GC-rich, show a high thermodynamic propensity for intra-strand stem loop formation (Figure 2C), a structural feature that enhances binding affinity to APOBEC1 (28). However, we were aware that unless the editing activity of APOBEC1 is regulated, allocation of the edited pool of notch-1 transcript and the associated degradation rate cannot be precisely controlled at G1 phase.

Specific features of the edited transcript enabled us to disclose a tripartite mechanism by which the activity of APOBEC1 is regulated at G0 phase. First, we noted that editing was not confined to a single nucleotide, rather several nucleotides in exon 25–27 were substituted in a pattern compatible with the reported hyper-editing mode of activity of APOBEC1 (29). The hyper-editing mode of APOBEC1 is activated upon over-expression of the protein (29). Profiling the expression of APOBEC1 revealed significant upregulation at G0 (Figure 2D), a finding that explains the APOBEC1-mediated hyper-editing of notch-1 transcript in this phase. Second, absence of the key NMD mediator UPF-1 at G0 uncouples NMD from editing and prevents rapid degradation of the edited transcript (Figure 2E). Third, we found that the APOBEC1-mediated hyper-editing affected nAS25 in a manner similar to the editing of notch-1 transcript (Figure 2F). Further, RNAi-mediated inhibition of nAS25 led to amplified editing of exons 25–27 of notch-1 transcript (Figure 2G). This finding suggested that nAS25 provides partial protection from RNA editing for notch-1 transcript, by hybridizing to the transcript and acting as a hybridised decoy target for APOBEC-1 (Figure 2H). We concluded that the tripartite mechanism for regulating the ratio of edited to non-edited notch-1 transcript consists of: (i) the amplified activity of APOBEC1 at G0 that defines the temporal window of hyper-editing; (ii) UPF1-mediated uncoupling of RNA editing and NMD (G0 vs. G1); and (iii) nAS25-mediated competitive titration of the editing activity of APOBEC1.

**Figure 3.**
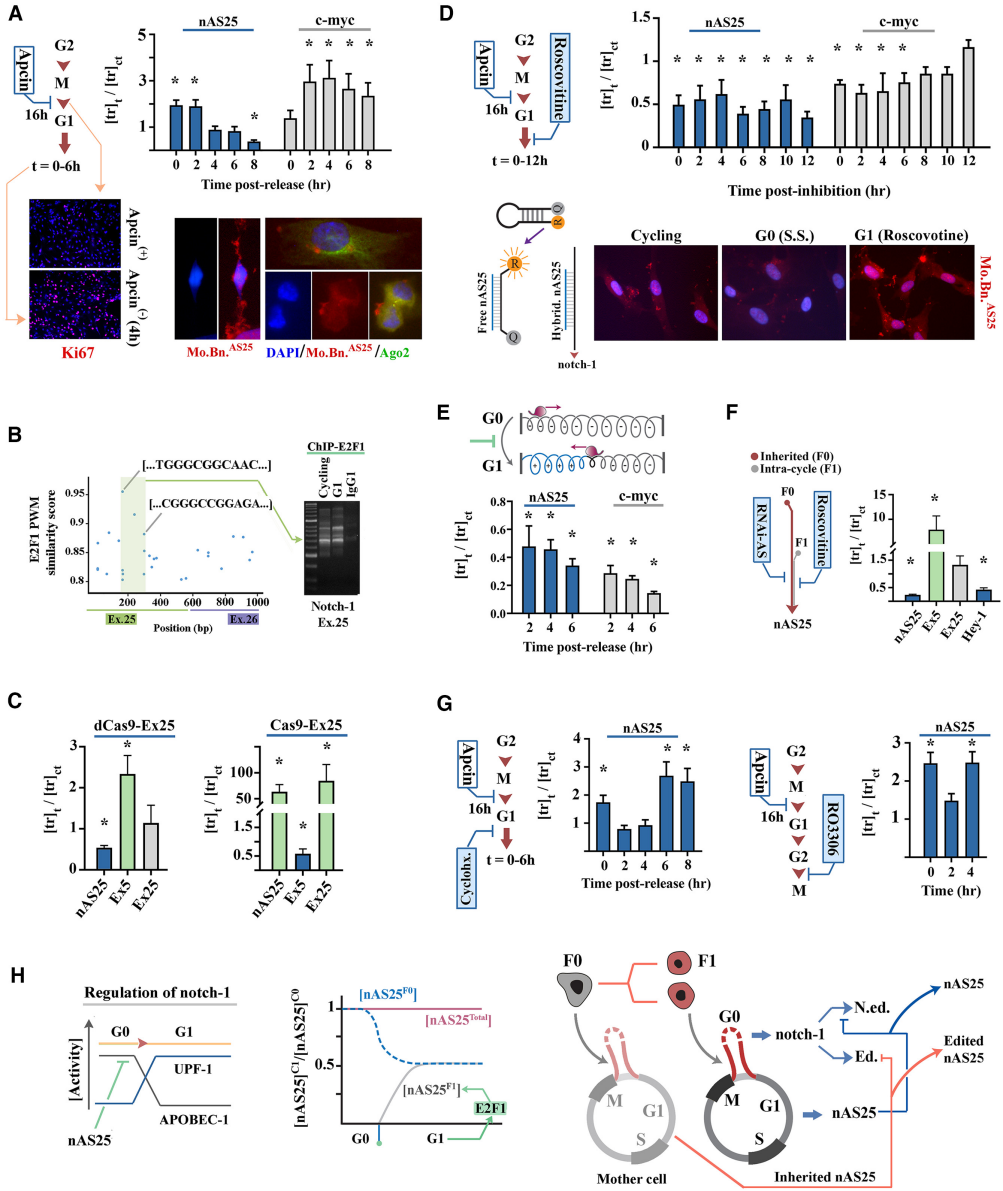
Cell cycle dynamics regulate transcription of nAS25. (**A**) Expression level of nAS25 upon progression from M to G1 phase. Expression of C-myc and ki-67, as reporters for cell cycle, increases upon progression to G1. Visualisation of the molecular beacon specific to nAS25 (Mo.Bn.^AS25^, top micrograph) shows inheritance of nAS25 by daughter cells during cytokinesis (bottom micrographs). * indicates two-tailed *P*-value < 0.01. (**B**) Scatter plot shows distribution of putative E2F1 binding sites in exons 25 and 26 of notch-1 locus. Subsequent ChIP analysis (right gel) confirmed binding of E2F1 to the highlighted region of exon 25 in cycling and G1 phase cells. (**C**) Expression level of nAS25, exon 5 and exon 25 of notch-1 after blocking the E2F1-binding site (in exon 25 as per text) using dCas9 and after cleavage of DNA upstream to this site. (**D**) Expression of nAS25 after pharmacological inhibition of Cdk2 using Roscovitine (cell cycle reporter: c-Myc). Application of Mo.Bn.^AS25^ to detect free nAS25 revealed higher expression of the antisense transcript in G1-arrested cells (Roscovitine^+^) relative to cycling and G0-arrested (S.S.) cells. (**E**) Expression of nAS25 at G1 subsequent to stabilisation of chromatin topology of G0-synchronised cells using TMP/UVA. (**F**) Expression of nAS25, exon 5, exon 25 and Hey-1 after simultaneous targeting of the inherited and intra-cycle nAS25. (**G**) Left bar plots show the level of nAS25 after cycloheximide-mediated inhibition of protein synthesis and the resultant lengthening of G1. Right bar plots show the level of nAS25 after inhibition of CDK-1 using RO3306 and the resultant arrest of cycling cells at G2. (**H**) The proposed model for allocation of degradable pool of notch-1 transcript at G0. ABPOBEC1-mediated editing of notch-1 transcript at G0 is prevented by competitive binding of nAS25 to the transcript. The edited pool of notch-1 is subsequently degraded at G1 via NMD and after activation of UPF-1. While ≈ 50% of the inherited nAS25 (nAS25^F0^) is edited and degraded, intra-cycle generation of the antisense transcript (nAS25^F1^) via E2F1 re-established the pre-G1 titer of nAS25 (nAS25^total^). The right schematic image presents a summary of the model regarding coupled activity of nAS25/notch-1. The inherited nAS25 protects full notch-1 at G0 by hybridizing to the transcript. The intracycle generation of nAS25 stops transcription of full notch-1 and is transmitted to daughter cells as a non-edited entity.

Evidence for involvement of nAS25 in regulating the G0-specific editing of notch-1 posed a temporal paradox; that is nAS25 is transcribed at G1 phase, only after completion of the editing activity of APOBEC1 (Figure 2C). Hence, we hypothesized that nAS25 generated in the mother cell provides protection for notch-1 transcript at G0 phase in daughter cells. In order to validate this hypothesis, we employed a strategy of mitotic arrest of cycling cells and G1-phase chase of nAS25. Cycling cells were reversibly synchronised at M phase by application of the APC inhibitor Apcin and were subsequently released into G1 phase by washing out the inhibitor (Supplementary Figure S2). We found that in transition to G1, the level of nAS25 in cycling cells was reduced by ≈ 2 fold (Figure 3A, Supplementary Figure S8). The decreased level of nAS25 concomitant with progression of cell cycle was consistent with degradation of the antisense transcript (Figure 3A). Knowing that nAS25 is transcribed at G1, detection of nAS25 at G0 in daughter cells suggested potential inheritance of the transcript from the mother cell. While application of a molecular beacon specific to nAS25 (Mo.Bn.^AS25^) provided preliminary evidence for the inheritance of nAS25 by daughter cells (Figure 3A), further validation of this conclusion required quantification of inherited nAS25 after blocking the intra-cycle synthesis of nAS25.

### nAS25 transmits information regarding the tempo of G1 phase of mother cell to daughter cells

Bidirectional transcription from exon 25 upon transition from G0 to G1 suggested that a G1-specific transcription factor activates a cryptic promoter in the latter exon. *In-silico* analysis of exon 25 revealed enrichment of *cis*-binding motifs with a high affinity for E2F1, the key driver of G1 phase dynamics (30) that binds to bidirectional promoters (31). Application of ChIP provided preliminary evidence that E2F1 is associated with exon 25 in G1 phase cells (Figure 3B, Supplementary Figure S9). To confirm the role of E2F1 in *cis*-activation of nAS25, we employed multiple techniques to block this proposed activation. Initially, we guided catalytically dead dCas9 to block the latter promoter using an sgRNA (Exon 25: C^161^-C^184^) that hybridises to a high affinity E2F1 binding motif (Exon 25: T^161^-C^171^, ≈96% homology to E2F1 PWM: MA0024.2, Figure 3B). Blocking this sequence resulted in reduction of nAS25 level by ≈ 46% and concomitant upregulation of exon 5 of notch-1 by ≈ 2.3 fold (Figure 3C). One explanation for the observed trend is that in the absence of antisense transcription, processivity of RNAP-II in the sense direction increases due to reduced positive supercoiling of DNA as per Figure 1C. Corroborating this notion, Cas9-mediated cleavage of DNA upstream to this site (intron 24: CCCGATCT…GTTGGTA) led to significant upregulation of nAS25/exon25 by uncoupling exon 25 from the upstream 5′-region (Figure 3c).

Further validation of the role of E2F1 in *cis*-activation of nAS25 was afforded by pharmacological inhibition of Cdk2 using Roscovitine (Seliciclib), a Cdk2 ATP-competitive inhibitor. Activity of Cdk2/Cyclin E is essential for phosphorylation of Retinoblastoma and release of E2F1. Roscovitine-treated cells progress into G1 due to continued activity of Cdk4/6/Cyclin D, but ultimate transition to S-phase is blocked owing to the absence of Cdk2/Cyclin E signaling and the resultant lack of free E2F1 (32). Cells were synchronised at M phase using Apcin (16h) and were then released into G1 by washing out Apcin and meanwhile adding fresh growth medium containing Roscovitine (20μM). We found that the level of nAS25 in Roscovitine-treated cells decreased by ≈ 50% (Figure 3D), closely reflecting the kinetics of nAS25 after blocking the E2F1 binding site using dCas9 (Figure 3C). Notably, tracking the non-hybridised nAS25 using Mo.Bn.^AS25^ revealed higher expression of the antisense transcript in Roscovitine-treated G1-arrested cells compared to the G0-arrested serum-starved cells (Figure 3D). This finding suggested that inherited nAS25 is either entirely hybridised to notch-1 or edited, both of which rendered the transcript undetectable by the molecular beacon. In contrast, the availability of free nAS25 provided further evidence of *de novo* transcription of the antisense transcript at G1 phase. We also applied Psoralen/UVA to stabilise chromatin topology of notch-1 locus in G0-synchronised cells and to inhibit antisense transcription by preventing the remodelling of chromatin topology upon transition to G1 phase (Figure 1C). In cells with stabilised chromatin topology (i.e. no intra-cycle generation of antisense transcript), the level of nAS25 was < 47% of the level in control cells. These results were in accord with findings from Roscovitine and dCas9 treatments, suggesting a mode of activity whereby ≈ 50% of the inherited nAS25 is edited at G0 and then degraded at G1, and meanwhile intra-cycle generation of nAS25 restores the level to the pre-G1 level. We also noted that simultaneous application of RNAi to target the inherited nAS25 and Roscovitine to block the intra-cycle generation of the antisense, reduced the level of nAS25 to 23% of titer in control cells (Figure 3E). We then asked if transient arrest at G1 would lead to accumulation of nAS25 via E2F1-dependent intra-cycle generation. Application of Cycloheximide to prolong G1 phase by suppressing protein synthesis led to accumulation of nAS25 (Figure 3G). Likewise, application of Crenigacestat, a selective Notch-1 inhibitor, to delay G1-S transition, increased the titer of nAS25 in G1 by ≈52% after 3–5 h (Supplementary Figure S10). As expected, synchronisation of cells at G2 phase by pharmacological inhibition of Cdk-1 (inhibitor: RO3306, 50 nM (33)) did not alter the level of nAS25 that could be transmitted to daughter cells (Figure 3G). While findings suggest that nAS25 can communicate cycling tempo of the mother cell to daughter cells in an E2F1-dependent manner (Figure 3H), it remained unclear whether the transmitted nAS25 could reprogram notch-1 level and G1 phase tempo of daughter cells to compensate for the altered dynamics of the mother cell.

### Regulation of notch-1 by inherited nAS25 reprograms the tempo of G1 phase in daughter cells

**Figure 4.**
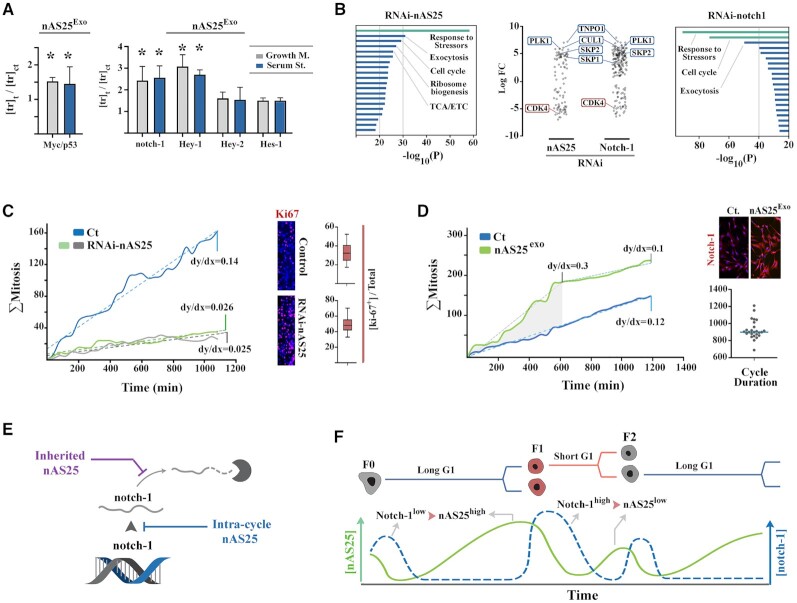
Cell cycle is effectively programmed by nAS25. (**A**) Increased ratio of C-myc (a positive mediator of cell cycle and downstream mediator of Notch-1) to p53 (negative regulator of cell cycle, not linked to Notch-1) was triggered by exogenous nAS25 (nAS25^Exo^), both in favorable (growth medium) and unfavorable (serum starved) conditions. Likewise, nAS25^Exo^ increased the level of notch-1 and its downstream mediator, hey-1. * indicates two-tailed *P*-value <0.01. (**B**) Graphs summarise GO enrichment analysis of RNA-seq profiles of cells upon RNAi-mediated suppression of notch-1 and of nAS25 (Analysis platform: Metascape). Scatter plots shows transcriptional profile of the GO cluster that regulates cell cycle. Note the similarity of outcomes instructed by direct RNAi-mediated inhibition of notch-1 and by indirect inhibition by RNAi-mediated depletion of nAS25. (**C**) Linear approximation of cumulative mitotic rate (dy/dx) in control cells and after RNAi-mediated inhibition of inherited nAS25. (**D**) Linear approximation of cumulative mitotic rate (dy/dx) in control cells and after exogenous amplification of inherited nAS25. (**E**) The schematic model shows calibration of notch-1 at G0 by inracycle generation of nAS25 that stops further transcription of notch-1 upon progression to G1. The inherited nAS25, on the other hand, protects that transcribed notch-1 at G0 and allocates the non-degradable pool of the transcript according to the cell cycle history of mother cell. (**F**) The proposed model for trangenerational coordination of cell cycle by simple harmonic oscillation of nAS25. The inherited nAS25 is initially degraded at G1 and is subsequently restored by intra-cycle E2F1-mediated transcription. Accordingly, notch-1^low^/nAS25^high^ profile of a G1^slow^ F0 cell will be followed by notch-1^high^/nAS25^low^ profile of G1^fast^ daughter cell.

To answer this question, we synthesized nAS25 and transfected the cycling cells (nAS25^exo^) to mimic amplification of nAS25 that is inherited from G1^slow^ mother cells. Amplification of nAS25 resulted in upregulation of notch-1 and its downstream mediator Hey-1, along with the pro-anabolic driver of cell cycle, c-Myc (Figure 4A). Notably, serum starvation did not affect nAS25^exo^-mediated upregulation of notch-1 and its downstream effectors (Figure 4A). This finding corroborated the notion that the fate of notch-1 transcript is uncoupled from stressors that occur in the same cycle. Rather, it is the cell cycle history of the mother cell, communicated via nAS25 (or nAS25^exo^), that determines the fate of the transcript. On the other hand, RNAi-mediated inhibition of nAS25, to mimic decreased inheritance of the antisense transcript from G1^fast^ mother cells, led to global transcriptional remodeling consistent with arrest at early G1 phase (Figure 4B). Upon direct RNAi-mediated inhibition of notch-1 or its indirect inhibition by RNAi-mediated depletion of nAS25, components of the SCF complex, that inhibits the cyclin-dependent kinase inhibitor p27, were upregulated and key drivers of G1 phase, including Cdk4, were downregulated (Figure 4B). Further analysis revealed that both RNAi-nAS25 and RNAi-notch1 cells were arrested in an ‘alert’ state (34), a transitional phase at early G1 that is characterised by upregulation of stress response and adaptation genes, consistent with the proposed role of notch-1 as a bistable regulator of G1 phase (35). We used single cell tracking by live imaging to validate the impact of nAS25 on the rate of proliferation of cycling cells. Cumulative graphs of mitotic events were generated as described previously (2,36) and the linear mitotic rate was calculated based on the slope of the least-squares regression line (dotted lines in Figure 4C, D) that optimally describes the data points. RNAi-mediated repression of nAS25 reduced the proliferation rate by ≈ 5.4 fold (Figure 4C). Amplification of nAS25 (nAS25^exo^) initially increased mitotic rate by 2.5 fold (Figure 4D). However, the linear mitotic rate of nAS25^exo^ was slightly lower than control cells after ≈ 600 min (Figure 4D). One plausible explanation for reduced linear mitotic rate after 600 min was that given the average length of cell cycle (≈900 min), a majority of F1 cells (>60%) would have completed cycle after the initial 600 min, giving rise to F2 cells that inherit a lower titer of nAS25 from nAS25^exo^/G1^fast^ F1 cells and hence the reduced mitotic rate.

## DISCUSSION

In conclusion, findings suggest that notch-1 is controlled by a bipartite mechanism. The temporal window for generation of the total pool of notch-1 transcripts is regulated by intracycle generation of nAS25 that stops transcription in the sense direction (Figure 4C). The inherited nAS25, on the other hand, inhibits RNA editing of the transcript and allocates the non-degradable pool of the transcript that will be translated at G1, according to the cell cycle history of the mother cell (Figure 4E). In consequence, slow harmonic oscillations of notch-1 and nAS25, with a periodicity that corresponds to the length of interphase, co-ordinate cell cycle dynamics between cellular generations (Figure 4F). In this mode of activity, G1 phase history of the mother cell is integrated into that of daughter cells at G0 phase to calibrate the titer of notch-1 transcript and the tempo of G1 phase in daughter cells (Figure 4F). The proposed model complements a long-established role of ligand/receptor interaction in activating the notch-1 signaling cascade by revealing a mechanism for regulating sensitivity of the signaling cascade by calibrating the availability of notch-1 transcript according to cell cycle history of the mother cell.

While the disclosed molecular platform for transgenerational coordination of cell cycle is novel, the theoretical basis for this phenomenon was established nearly four decades ago. Early attempts at explaining unresolved phenomena such as correlation of cell cycle duration between third-degree cell lineage (i.e., between mother-cousin pairs) (5–7) led to the prediction that an oscillatory system, slower than cyclins, must exist in metazoan systems to orchestrate cell cycle duration in cell pedigrees (5,37). Central to the latter argument was existence of a putative ‘compensation factor’ that is transmitted from mother cells to daughter cells to account for the long cell-cycle duration of the mother cell (6). The inheritance mode of nAS25 and the simple harmonic oscillation of notch-1/nAS25 with a periodicity that corresponds to the length of interphase, fulfill the assumptions of the theoretical platform for coordinated cycling in cell lineages. Particularly, the simple stoichiometric topology of notch signaling cascade, whereby signaling output is not amplified, is critical to communicate a high-fidelity undistorted history of the mother cell cycle into daughter cells. We predict that the disclosed mechanism for transgenerational adaptation of cell cycle safeguards against random oscillations of cell cycle and enhances robustness of development of multicellular organisms.

The demonstrated role of nAS25 in programming the fate of notch-1 transcript bears close similarity to the role of natural antisense transcription in regulating activity of the circadian clock (13). Mosig et al. demonstrated that discordant transcription of sense and antisense transcripts from Period2 locus leads to repression of Per2 upon transcription of the *Cis*-natural antisense transcript, a mechanism by which the level of Per2 is maintained within the oscillatory range (13). This is similar to the function nAS25 in repressing transcription of notch-1 upon progression into G1 phase of cell cycle. Likewise, timing of entry into meiosis in *Saccharomyces cerevisiae* is programmed by an antisense transcript that binds to a highly conserved internal regions of the IME4 locus and blocks transcriptional elongation (38). Apart from transcription-level interference, sense and antisense transcripts could interact after transcription. Faghihi et al. demonstrated that BACE1-antisense prevents miRNA-induced targeting of BACE1 mRNA by masking the binding site for miR-485–5p (39). The activity of BACE1-AS in masking the miRNA binding site on BACE1 transcripts bears close resemblance to nAS25-mediated masking of exon 25 of notch-1. In conclusion, it becomes apparent that nAS25 and notch-1 interact at multiple levels (including transcriptional control and fate of transcript) and each interaction contributes to regulation of transcript availability in an exclusive and non-redundant manner.

## DATA AVAILABILITY

The published article includes all datasets generated or analyzed during this study. The RNA-seq data described in this study are deposited to the Gene Expression Omnibus (GEO) repository. The accession number for the RNA-seq data reported in this paper is GEO: GSE168092. The Capture-seq data described in this study are deposited to the Sequence Read Archive (SRA). The accession number for the Capture-seq data reported in this paper is SRA: PRJNA705559.

## Supplementary Material

gkab800_Supplemental_File
