# Supplementary material for: The fate of notch-1 transcript is linked to cell cycle dynamics by activity of a natural antisense transcript
Source: Nucleic Acids Res. 2021 Sep 14;49(18):10419–30. doi: 10.1093/nar/gkab800 (PMC8501981; doi:10.1093/nar/gkab800)
Supplement: gkab800_Supplemental_File [file gkab800_supplemental_file.pdf]

Supplementary Files

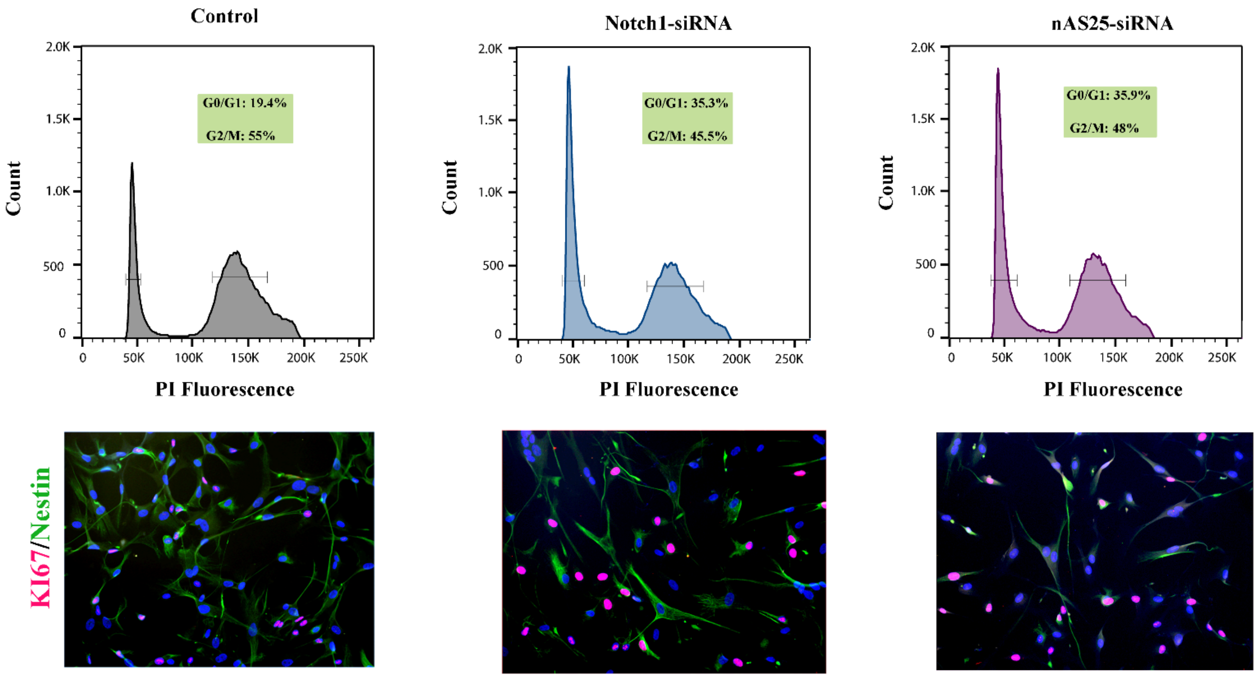

Supplementary Figure S1. Top images show flow cytometry cell cycle analysis using propidium iodide DNA staining. Note enrichment of G1 phase population subsequent to siRNA-mediated knockdown of notch-1 and similar finding subsequent to siRNA-mediated inhibition of nAS25. Bottom micrographs show Ki-67 immunoreactivity in control cycling cells and after siRNA-mediated knockdown of notch-1 and nAS25.

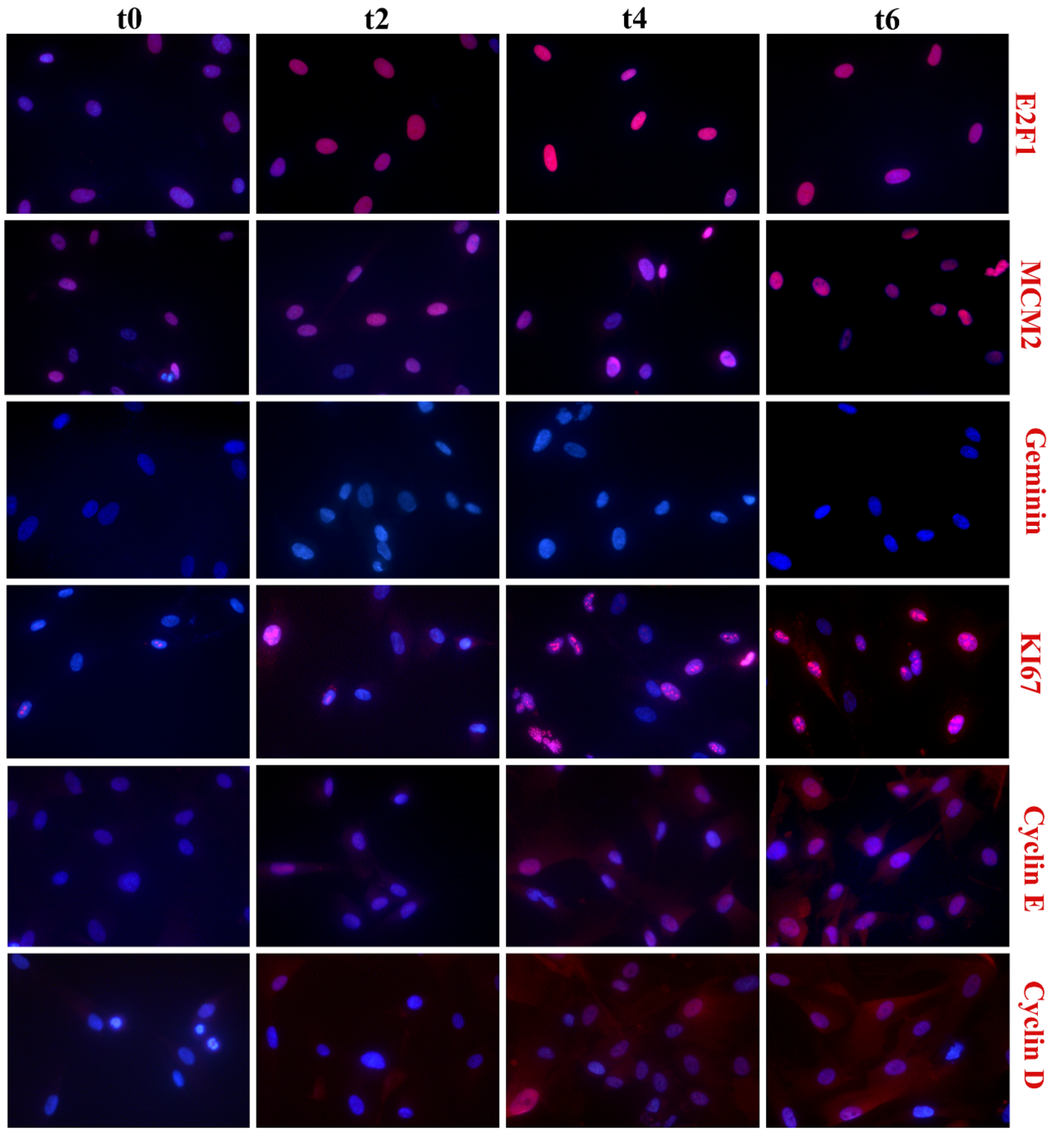

Supplementary Figure S2. Immunohistochemical fingerprinting of cell cycle-related proteins in Apcin-chase methods. Note that t=4h accommodates a KI67^{high}/Cyclin-D^{high}/Cyclin-E^{high}/Geminin^{-} profile that is consistent with transition to G1.

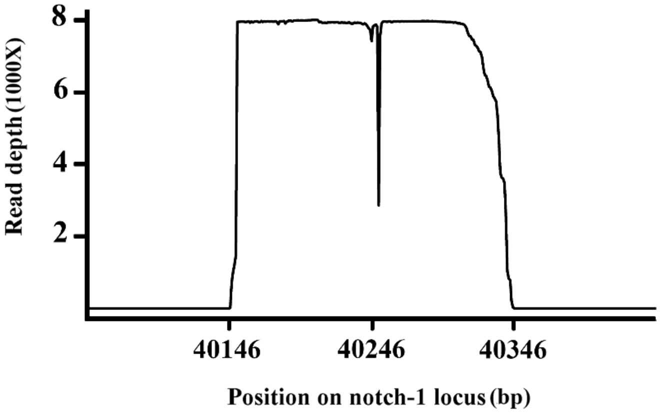

Supplementary Figure S3. Capture-seq profile of nAS25 isolated from the serum-starved cells shows the position of the antisense transcript within exon 25 of notch-1.

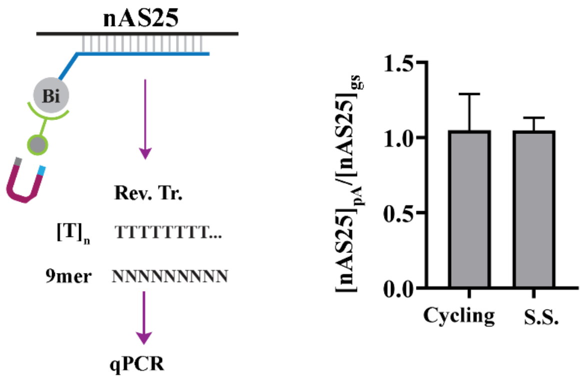

Supplementary Figure S4. Bar plots show the analysis by qPCR of [nAS25] that is captured by nAS25-specific oligo and reverse transcribed using poly-dT (i.e. $[nAS25]_{pA}$) and nAS25-specific primers (i.e. $[nAS25]_{gs}$). Normalised ratio of $[nAS25]_{pA}/[nAS25]_{gs}$ corroborates the Capture-seq and RNA-seq results that majority of nAS25 transcript are polyadenylated.

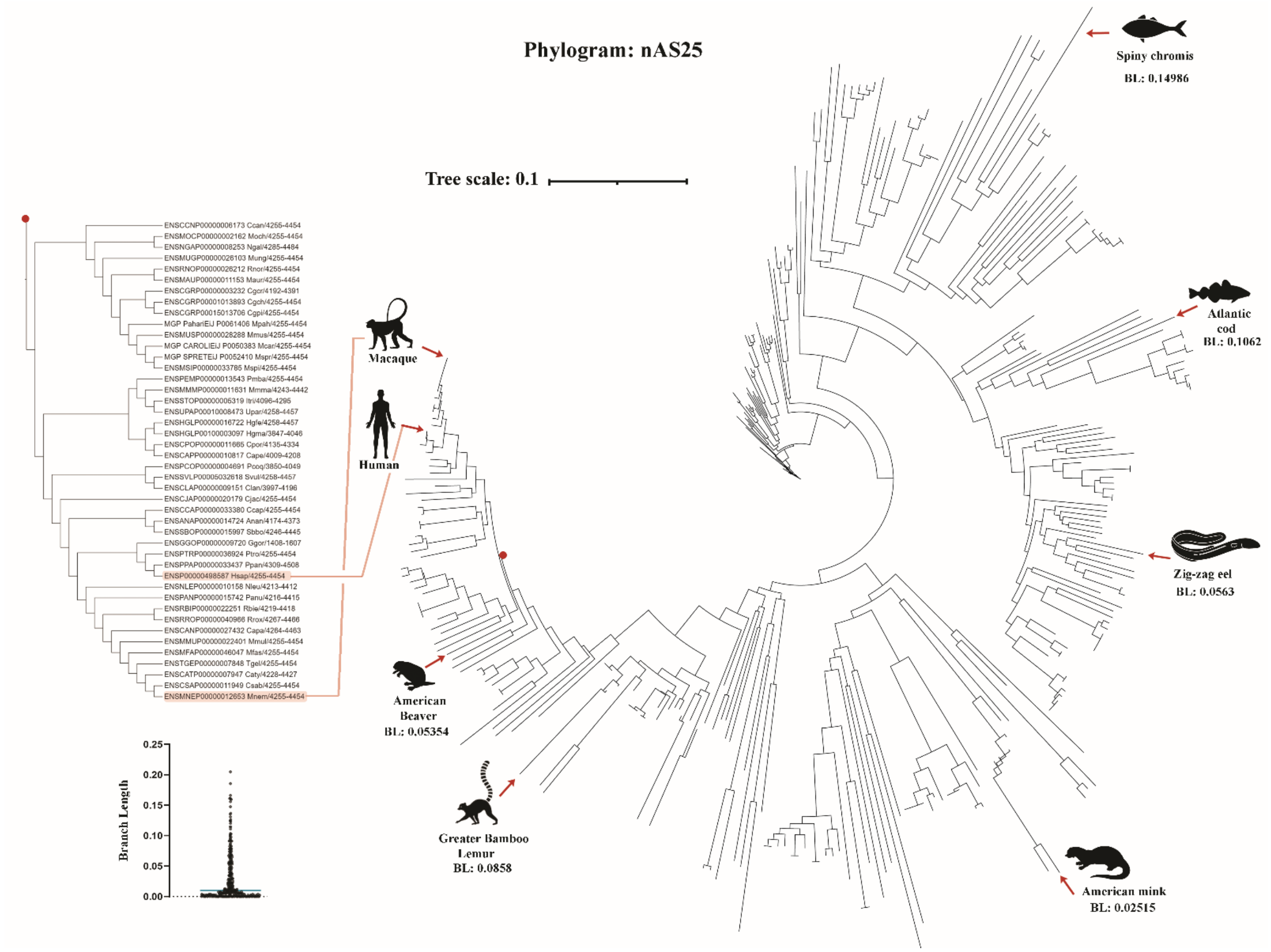

Supplementary Figure S5. Phylogram shows the most parsimonious estimate of phylogeny (n=332 metazoan species) for the region corresponding to A^{241}: A^{440} of exon 25 of human notch-1. Sequence alignment and estimation of branch length (BL) were calculated using Clustal Omega. Linear cladogram (left) provides an expanded view of the region of the circular phylogram entailing human and other primates and marked by a red circle. The scatter plot shows distribution of branch length for the studied species. Note the minimal divergence of the region corresponding to A^{241}: A^{440} of exon 25 of human notch-1 in major clades of metazoan species.

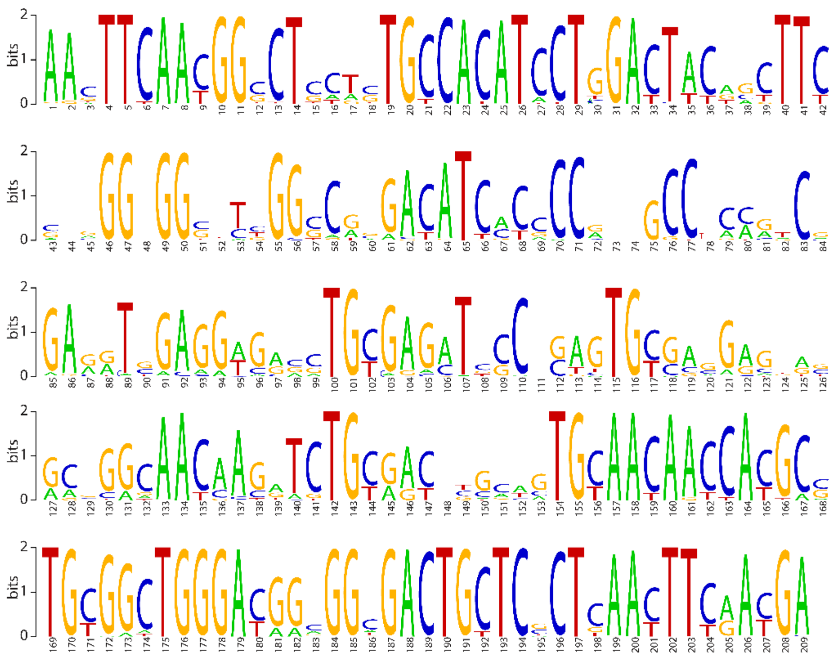

Supplementary Figure S6. Sequence logo shows high conservation of the region corresponding to A^{241}: A^{440} of exon 25 of human notch-1 (5′ region: top, 3′ region: bottom) in major clades of metazoan species (n=332 metazoan species). Note the high conservation of the 5′ and 3′ sequences corresponding to the transcription initiation sit (bottom sequence logo) and polyadenylation signal (top sequence logo), respectively.

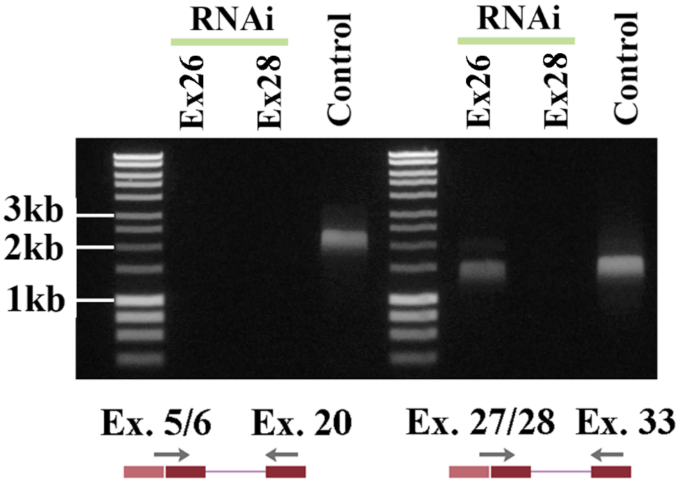

Supplementary Figure S7. Gel shows notch-1 amplicons that span exons 5-20 and exons 27-33, after RNAi-mediated targeting of exon 26 and exon 28 of notch-1. Note that the amplicon corresponding to exons 27-33 remain unaffected by RNAi-mediated targeting of exon 26. This finding indicates that the truncated noncoding notch-1 transcript and the full notch transcript exist as two separate entities.

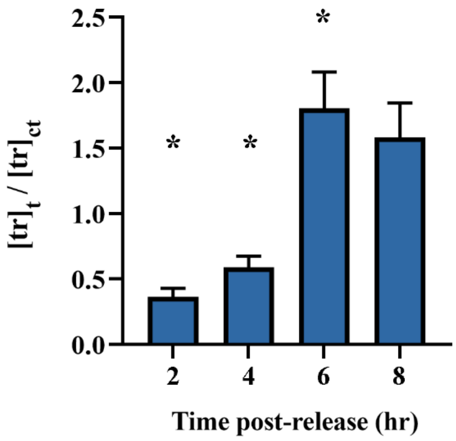

Supplementary Figure S8. Expression level of nAS25 in G1 phase cycling cells normalised to G0-synchronised cells (serum starvation for 24h). * indicates two-tailed p-value<0.01.

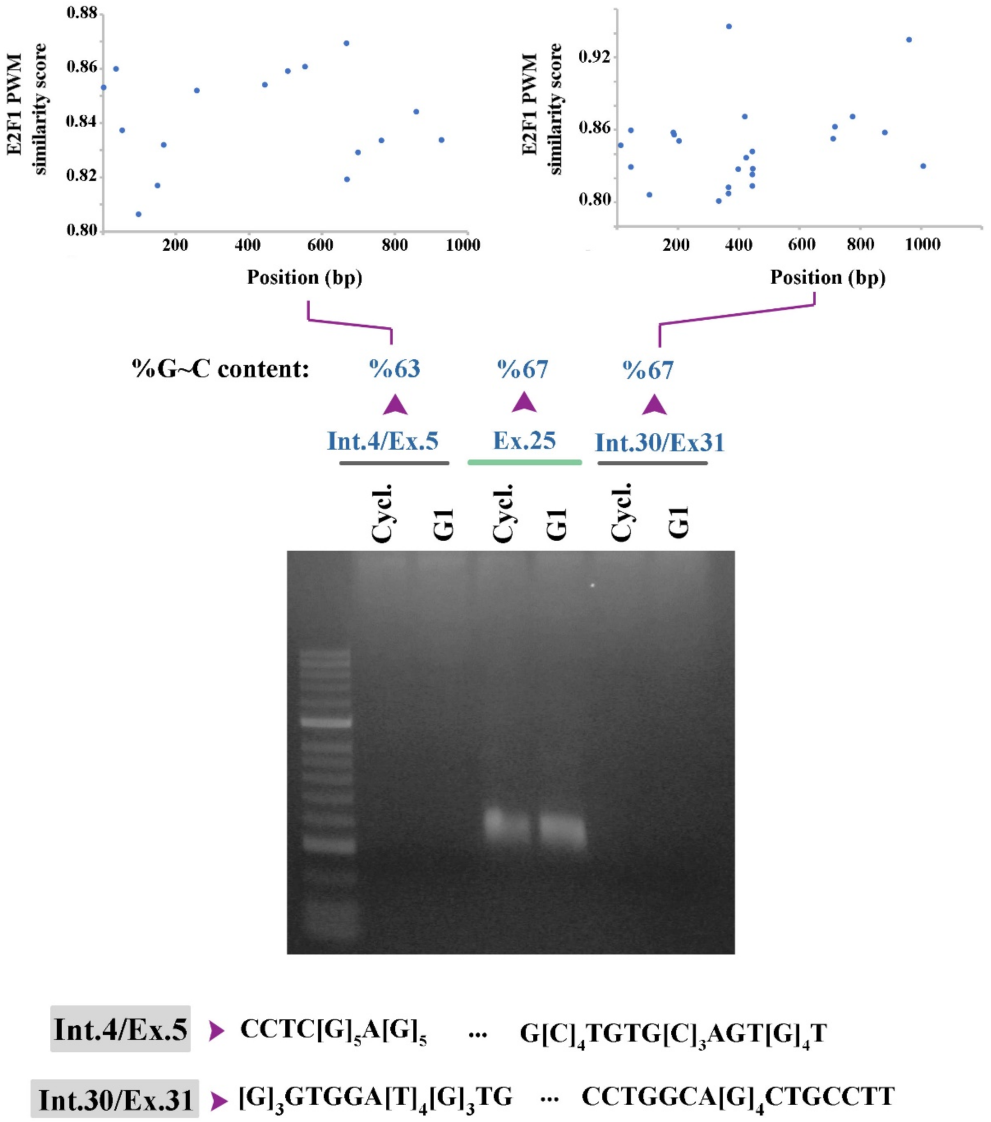

Supplementary Figure S9. Scatter plot shows distribution of putative E2F1 binding sites in GC-rich regions encoded by intron 4/exons 5 and intron 30/exon 31 of notch-1 locus. Subsequent ChIP analysis (bottom gel) confirmed that E2F1 does not bind to these regions as opposed to its association with exon 25.

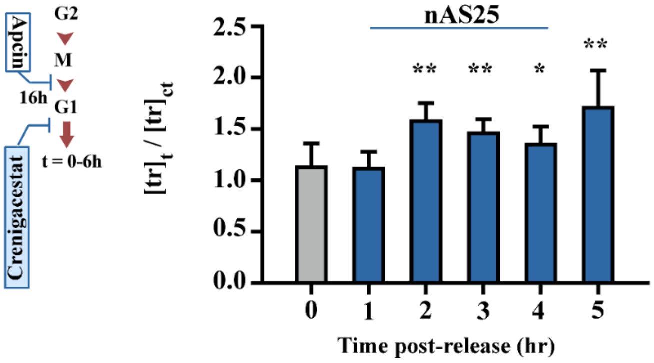

Supplementary Figure S10. Bar plot shows the level of nAS25 after application of Crenigacestat, a selective Notch-1 inhibitor, to delay G1-S transition. $^{**}p<0.001$ and $^{*}p<0.05$.

Supplementary Table S1. Phylogenetic analysis of conserved D-box sequence in UPF-1 protein.

YMR080C_Scer/1-971	PCAQ---TKNANWDTDQWQPLIEDRQLLSWVAEQPTEEEKLKARLITPSQISKLEAKWR-	206
ENSPCAP00000012158_Pcap/1-1115	PCASQSSLKDINWDSSQWQPLIQDRCFLSWLVKIPSEQEQLRARQITAQQINKLEELWK-	268
ENSPVAP00000003004_Pvam/1-1119	PCASQSSLKDINWDSSQWQPLIQDRCFLSWLVKIPSEQEQLRARQITAQQINKLEELWK-	271
ENSCSAVP00000011178_Csav/1-1093	PCANQTSLKDMNWDAAQWQPLIQDRCFLSWLVKIPSEEEQLRARQITAQQINKLEELWK-	253
FBpp0073433_Dmel/1-1180	PCAAQNSLKDMNWDQEQWKPLIADRCFLAWLVKQPSEQGQLRARQISAAQINKLEELWK-	247
ENSCINP00000031943_Cint/1-836	PCANQTSLKDMNWDASQWQPLIQDRCFLSWLVKIPSEEEQLRARQITAQQINKLEELWK-	238
ENSCCRP00000096280_Ccar/1-730	PCASQSSLKDINWDSSQWQPLIQDRCFLSWLVKIPSEQEQLRARQITAQQINKLEELWK-	249
ENSCCRP00000065172_Ccar/1-730	PCASQSSLKDINWDSSQWQPLIQDRCFLSWLVKIPSEQEQLRARQITAQQINKLEELWK-	249
ENSPMAP00000001338_Pmar/1-1106	PCASQSSLKDMNWDATQWQPLIQDRCFLPWLVKIPSEQEQLRARQITAQQINKLEELWK-	254
ENSEBUP00000016049_Ebur/1-623	PCASQSSLKDMNWDASQWQPLIQDRCFLPWLVKVPSEQEQLRARQITAQQINKLEELWK-	257
ENSSGRP00000007313_Sgra/1-946	PCASQSSLKDINWDSSQWQPLIQDRCFLSWLVKIPSEQEQLRARQITAQQINKLEELWK-	249
ENSZALP00000008603_Zalb/1-589	PCASQSSLKDINWDSSQWQPLIQDRCFLSWLVKIPSEQEQLRARQITAQQINKLEELWK-	246
ENSMMMP00000002826_Mmma/1-1074	PCASQSSLKDINWDSSQWQPLIQDRCFLSWLVKIPSEQEQLRARQITAQQINKLEELWK-	260
ENSCCRP00000013506_Ccar/1-996	PCASQSSLKDINWDSSQWQPLIQDRCFLSWLVKIPSEQEQLRARQITAQQINKLEELWK-	198
ENSOKIP00005078820_Okis/1-1101	PCASQSSLKDINWDSSQWQPLIQDRCFLSWLVKIPSEQEQLRARQITAQQINKLEELWK-	273
ENSPMJP00000017881_Pmaj/1-928	PCASQSSLKDINWDSSQWQPLIQDRCFLSWLVKIPSEQEQLRARQITAQQINKLEELWK-	269
ENSCPIP00010007423_Cpic/1-646	PCASQSSLKDINWDSSQWQPLIQDRCFLSWLVKIPSEQEQLRARQITAQQINKLEELWK-	207
ENSHBUP00000012883_Hbur/1-903	PCASQSSLKDINWDSSQWQPLIQDRCFLSWLVKIPSEQEQLRARQITAQQINKLEELWK-	234
ENSSRHP00000093447_Srhi/1-1058	PCASQSSLKDINWDSSQWQPLIQDRCFLSWLVKIPSEQEQLRARQITAQQINKLEELWK-	249
ENSOPRP00000012816_Opri/1-1033	PCASQSSLKDINWDSSQWQPLIQDRCFLSWLVKIPSEQEQLRARQITAQQINKLEELWK-	193
ENSXCOP00000020391_Xcou/1-1086	PCASQSSLKDINWDSSQWQPLIQDRCFLSWLVKIPSEQEQLRARQITAQQINKLEELWK-	249
ENSXMAP00000001707_Xmac/1-1084	PCASQSSLKDINWDSSQWQPLIQDRCFLSWLVKIPSEQEQLRARQITAQQINKLEELWK-	249
ENSPREP00000014749_Pret/1-1109	PCASQSSLKDINWDSSQWQPLIQDRCFLSWLVKIPSEQEQLRARQITAQQINKLEELWK-	251
ENSPMEP00000009530_Pmex/1-1094	PCASQSSLKDINWDSSQWQPLIQDRCFLSWLVKIPSEQEQLRARQITAQQINKLEELWK-	249
ENSPFOP00000004560_Pfor/1-1107	PCASQSSLKDINWDSSQWQPLIQDRCFLSWLVKIPSEQEQLRARQITAQQINKLEELWK-	249
ENSPLAP00000014427_Plat/1-1107	PCASQSSLKDINWDSSQWQPLIQDRCFLSWLVKIPSEQEQLRARQITAQQINKLEELWK-	249
ENSCSEP00000027006_Csem/1-1075	PCASQSSLKDINWDSSQWQPLIQDRCFLSWLVKIPSEQEQLRARQITAQQINKLEELWK-	247
ENSCCRP00015004770_Cchu/1-995	PCASQSSLKDINWDSSQWQPLIQDRCFLSWLVKIPSEQEQLRARQITAQQINKLEELWK-	255
ENSHHUP00000022906_Hhuc/1-1094	PCASQSSLKDINWDSSQWQPLIQDRCFLSWLVKIPSEQEQLRARQITAQQINKLEELWK-	248
ENSELUP00000040396_Eluc/1-1128	PCASQSSLKDINWDSSQWQPLIQDRCFLSWLVKIPSEQEQLRARQITAQQINKLEELWK-	263
ENSOTSP00005056657_Otsh/1-1101	PCASQSSLKDINWDSSQWQPLIQDRCFLSWLVKIPSEQEQLRARQITAQQINKLEELWK-	248
ENSOMYP00000104090_Omyk/1-1101	PCASQSSLKDINWDSSQWQPLIQDRCFLSWLVKIPSEQEQLRARQITAQQINKLEELWK-	248
ENSOKIP00005117028_Okis/1-1101	PCASQSSLKDINWDSSQWQPLIQDRCFLSWLVKIPSEQEQLRARQITAQQINKLEELWK-	248
ENSSSAP00000109424_Ssal/1-1101	PCASQSSLKDINWDSSQWQPLIQDRCFLSWLVKIPSEQEQLRARQITAQQINKLEELWK-	248
ENSSTUP00000105246_Stru/1-1101	PCASQSSLKDINWDSSQWQPLIQDRCFLSWLVKIPSEQEQLRARQITAQQINKLEELWK-	248
ENSHHUP00000064237_Hhuc/1-1112	PCASQSSLKDINWDSSQWQPLIQDRCFLSWLVKIPSEQEQLRARQITAQQINKLEELWK-	248
ENSOMYP00000004009_Omyk/1-1121	PCASQSSLKDINWDSSQWQPLIQDRCFLSWLVKIPSEQEQLRARQITAQQINKLEELWK-	268
ENSOTSP00005068575_Otsh/1-1114	PCASQSSLKDINWDSSQWQPLIQDRCFLSWLVKIPSEQEQLRARQITAQQINKLEELWK-	261
ENSSSAP00000098228_Ssal/1-1112	PCASQSSLKDINWDSSQWQPLIQDRCFLSWLVKIPSEQEQLRARQITAQQINKLEELWK-	259
ENSSTUP00000101695_Stru/1-1112	PCASQSSLKDINWDSSQWQPLIQDRCFLSWLVKIPSEQEQLRARQITAQQINKLEELWK-	259
ENSPMGP00000021401_Pmag/1-1100	PCASQSSLKDINWDSSQWQPLIQDRCFLSWLVKIPSEHEQLRARQITAQQINKLEELWK-	249
ENSNMLP00000016848_Nmel/1-1127	PCASQSSLKDINWDSSQWQPLIQDRCFLSWLVKIPSEHEQLRARQITAQQINKLEELWK-	273
ENSFHEP00000005383_Fhet/1-1110	PCASQSSLKDINWDSSQWQPLIQDRCFLSWLVKIPSEQEQLRARQITAQQINKLEELWK-	255
ENSCCRP00020076389_Cche/1-1011	PCASQSSLKDINWDSSQWQPLIQDRCFLSWLVKIPSEQEQLRARQITAQQINKLEELWK-	255
ENSTNIP00000014011_Tnig/1-1126	PCASQSSLKDINWDSSQWQPLIQDRCFLSWLVKIPSEQEQLRARQITAQQINKLEELWKG	250
ENSTRUP00000000308_Trub/1-1111	PCASQSSLKDINWDSSQWQPLIQDRCFLSWLVKIPSEQEQLRARQITAQQINKLEELWK-	247
ENSGWIP00000022221_Gwil/1-1146	PCASQSSLKDINWDSSQWQPLIQDRCFLSWLVKIPSEQEQLRARQIAAQQINKLEELWK-	249
ENSSPAP00000000003_Spar/1-1076	PCASQSSLKDINWDSSQWQPLIQDRCFLSWLVKIPSEQEQLRARQITAQQINKLEELWK-	229
ENSNBRP00000000837_Nbri/1-1087	PCASQSSLKDINWDSSQWQPLIQDRCFLSWLVKIPSEQEQLRARQITAQQINKLEELWK-	249
ENSPRNP00000048129_Pran/1-1112	PCASQSSLKDINWDSSQWQPLIQDRCFLSWLVKIPSEQEQLRARQITAQQINKLEELWK-	249
ENSSFAP00005040056_Sfas/1-1112	PCASQSSLKDINWDSSQWQPLIQDRCFLSWLVKIPSEQEQLRARQITAQQINKLEELWK-	249
ENSAOCP00000013856_Aoce/1-1113	PCASQSSLKDINWDSSQWQPLIQDRCFLSWLVKIPSEQEQLRARQITAQQINKLEELWK-	249
ENSAPEP00000023048_Aper/1-1113	PCASQSSLKDINWDSSQWQPLIQDRCFLSWLVKIPSEQEQLRARQITAQQINKLEELWK-	249
ENSAPOP00000020196_Apol/1-1113	PCASQSSLKDINWDSSQWQPLIQDRCFLSWLVKIPSEQEQLRARQITAQQINKLEELWK-	249
ENSACIP00000025783_Acit/1-1096	PCASQSSLKDINWDSSQWQPLIQDRCFLSWLVKIPSEQEQLRARQITAQQINKLEELWK-	249
ENSMZEP00005004537_Mzeb/1-1109	PCASQSSLKDINWDSSQWQPLIQDRCFLSWLVKIPSEQEQLRARQITAQQINKLEELWK-	249
ENSPNYP00000004833_Pnye/1-1109	PCASQSSLKDINWDSSQWQPLIQDRCFLSWLVKIPSEQEQLRARQITAQQINKLEELWK-	249
ENSACLP00000010541_Acal/1-1096	PCASQSSLKDINWDSSQWQPLIQDRCFLSWLVKIPSEQEQLRARQITAQQINKLEELWK-	249
ENSONIP00000013137_Onil/1-1109	PCASQSSLKDINWDSSQWQPLIQDRCFLSWLVKIPSEQEQLRARQITAQQINKLEELWK-	249
ENSOABP00000000157_Oaur/1-1109	PCASQSSLKDINWDSSQWQPLIQDRCFLSWLVKIPSEQEQLRARQITAQQINKLEELWK-	249
ENSDLAP00005012646_Dlab/1-1123	PCASQSSLKDINWDSSQWQPLIQDRCFLSWLVKIPSEQEQLRARQITAQQINKLEELWK-	281
ENSMMOP00000010211_Mmol/1-1091	PCASQSSLKDINWDSSQWQPLIQDRCFLSWLVKIPSEQEQLRARQIAAQQINKLEELWK-	249
ENSLBEP00000019395_Lber/1-1113	PCASQSSLKDINWDSSQWQPLIQDRCFLSWLVKIPSEQEQLRARQITAQQINKLEELWK-	249
ENSLCRP00005064618_Lcro/1-1108	PCASQSSLKDINWDSSQWQPLIQDRCFLSWLVKIPSEQEQLRARQITAQQINKLEELWK-	249
ENSSAUP00010014918_Saur/1-1113	PCASQSSLKDINWDSSQWQPLIQDRCFLSWLVKIPSEQEQLRARQITAQQINKLEELWK-	249
ENSCLMP00005018723_Clum/1-1112	PCASQSSLKDINWDSSQWQPLIQDRCFLSWLVKIPSEQEQLRARQITAQQINKLEELWK-	279
ENSGACP00000020456_Gacu/1-1115	PCASQSSLKDINWDSSQWQPLIQDRCFLSWLVKIPSEQEQLRARQITAQQINKLEELWK-	247
ENSSORP00005044495_Sorb/1-1127	PCASQSSLKDINWDSSQWQPLIQDRCFLSWLVKIPSEQEQLRARQITAQQINKLEELWK-	274
ENSSMAP00000008250_Smax/1-1113	PCASQSSLKDINWDSSQWQPLIQDRCFLSWLVKIPSEQEQLRARQITAQQINKLEELWK-	249
ENSCGOP00000033482_Cgob/1-1112	PCASQSSLKDINWDSSQWQPLIQDRCFLSWLVKIPSEQEQLRARQITAQQINKLEELWK-	249

```
ENSBSLP00000056310_Bspl/1-1111  PCASQSSLKDINWDSSQWQPLIQDRCFLSWLVKIPSEQEQLRARQITAQQINKLEELWK- 249
ENSSLUP00000008937_Sluc/1-1114  PCASQSSLKDINWDSSQWQPLIQDRCFLSWLVKIPSEQEQLRARQITAQQINKLEELWK- 249
ENSMMDP00005035276_Mmur/1-1116  PCASQSSLKDINWDSSQWQPLIQDRCFLSWLVKIPSEQEQLRARQITAQQINKLEELWK- 249
ENSATEP00000025816_Ates/1-1115  PCASQSSLKDINWDSSQWQPLIQDRCFLSWLVKIPSEQEQLRARQITAQQINKLEELWK- 249
ENSMAMP00000009272_Marm/1-1115  PCASQSSLKDINWDSSQWQPLIQDRCFLSWLVKIPSEQEQLRARQITAQQINKLEELWK- 249
ENSMALP00000000226_Malb/1-1075  PCASQSSLKDINWDSSQWQPLIQDRCFLSWLVKIPSEQEQLRARQITAQQINKLEELWK- 249
ENSENLP00000022966_Enau/1-1114  PCASQSSLKDINWDSSQWQPLIQDRCFLSWLVKIPSEQEQLRARQITAQQINKLEELWK- 249
ENSLCAP00010046411_Lcal/1-1115  PCASQSSLKDINWDSSQWQPLIQDRCFLSWLVKIPSEQEQLRARQITAQQINKLEELWK- 249
ENSSLDP00000026875_Sldo/1-1105  PCASQSSLKDINWDSSQWQPLIQDRCFLSWLVKIPSEQEQLRARQITAQQINKLEELWK- 249
ENSSDUP00000018286_Sdum/1-1115  PCASQSSLKDINWDSSQWQPLIQDRCFLSWLVKIPSEQEQLRARQITAQQINKLEELWK- 249
ENSDLAP00005046214_Dlab/1-1136  PCASQSSLKDINWDSSQWQPLIQDRCFLSWLVKIPSEQEQLRARQITAQQINKLEELWK- 271
ENSFHEP00000030731_Fhet/1-991   PCASQSSLKDINWDSSQWQPLIQDRCFLSWLVKIPSEQEQLRARQITAQQINKLEELWK- 214
ENSGWIP00000054730_Gwil/1-1137  PCASQSSLKDINWDSSQWQPLIQDRCFLSWLVKIPSEQEQLRARQITAQQINKLEELWK- 264
ENSTNIP00000002898_Tnig/1-1113  PCASQSSLKDINWDSSQWQPLIQDRCFLSWLVKIPSEQEQLRARQIAAQQINKLEELWK- 251
ENSHBUP00000007395_Hbur/1-1004  PCASQSSLKDINWDSSQWQPLIQDRCFLSWLVKIPSEQEQLRARQITAQQINKLEELWK- 251
ENSLSDP00000009944_Lsdo/1-1106  PCASQSSLKDINWDSSQWQPLIQDRCFLSWLVKIPSEQEQLRARQITAQQINKLEELWK- 267
ENSOANP00000051835_Oana/1-1099  PCASQSSLKDINWDSSQWQPLIQDRCFLSWLVKIPLEQEQLRARQITPQQMNKLEELWK- 252
ENSORLP00015009336_Olhs/1-1112  PCASQSSLKDINWDSSQWQPLIQDRCFLSWLVKIPSEQEQLRARQITAQQINKLEELWK- 252
ENSPKIP00000021830_Pkin/1-1039  PCASQSSLKDINWDSSQWQPLIQDRCFLSWLVKIPSEQEQLRARQITAQQINKLEELWK- 251
ENSLLTP00000017680_Llat/1-942   PCASQSSLKDINWDSSQWQPLIQDRCFLSWLVKIPSEQEQLRARQITAQQINKLEELWK- 268
ENSGMOP00000008081_Gmor/1-1107  PCASQSSLKDINWDSSQWQPLIQDRCFLSWLVKIPSEQEQLRARQITAQQINKLEELWK- 251
ENSTRUP00000012294_Trub/1-1118  PCASQSSLKDINWDSSQWQPLIQDRCFLSWLVKIPSEQEQLRARQITAQQINKLEELWK- 260
ENSOSIP00000042146_Osin/1-1122  PCASQSSLKDINWDSSQWQPLIQDRCFLSWLVKIPSEQEQLRARQITAQQINKLEELWK- 267
ENSORLP00020013752_Olhn/1-1102  PCASQSSLKDINWDSSQWQPLIQDRCFLSWLVKIPSEQEQLRARQITAQQINKLEELWK- 252
ENSORLP00000010622_Olat/1-1101  PCASQSSLKDINWDSSQWQPLIQDRCFLSWLVKIPSEQEQLRARQITAQQINKLEELWK- 251
ENSOMEP00000023512_Omel/1-1101  PCASQSSLKDINWDSSQWQPLIQDRCFLSWLVKIPSEQEQLRARQITAQQINKLEELWK- 251
ENSOJAP00000022740_Ojav/1-1101  PCASQSSLKDINWDSSQWQPLIQDRCFLSWLVKIPSEQEQLRARQITAQQINKLEELWK- 251
ENSSFAP00005015698_Sfas/1-1100  PCASQSSLKDINWDSSQWQPLIQDRCFLSWLVKIPSEQEQLRARQITAQQINKLEELWK- 251
ENSMMDP00005027456_Mmur/1-1102  PCASQSSLKDINWDSSQWQPLIQDRCFLSWLVKIPSEQEQLRARQITAQQINKLEELWK- 251
ENSMALP00000026074_Malb/1-1105  PCASQSSLKDINWDSSLWQPLIQDRCFLSWLVKIPSEQEQLRARQITAQQINKLEELWK- 251
ENSNFUP00015045546_Nfur/1-1123  PCASQSSLKDINWDSSQWQPLIQDRCFLSWLVKIPSEQEQLRARQITAQQINKLEELWK- 277
ENSKMAP00000006602_Kmar/1-1101  PCASQSSLKDINWDSSQWQPLIQDRCFLSWLVKIPSEQEQLRARQITAQQINKLEELWK- 251
ENSCVAP00000025589_Cvar/1-1102  PCASQSSLKDINWDSSQWQPLIQDRCFLSWLVKIPSEQEQLRARQITAQQINKLEELWK- 251
ENSPREP00000016656_Pret/1-1097  PCASQSSLKDINWDSSQWQPLIQDRCFLSWLVKIPSEQEQLRARQITAQQINKLEELWK- 251
ENSXCOP00000007480_Xcou/1-1028  PCASQSSLKDINWDSSQWQPLIQDRCFLSWLVKIPSEQEQLRARQITAQQINKLEELWK- 182
ENSXMAP00000002552_Xmac/1-1098  PCASQSSLKDINWDSSQWQPLIQDRCFLSWLVKIPSEQEQLRARQITAQQINKLEELWK- 251
ENSPFOP00000000758_Pfor/1-1097  PCASQSSLKDINWDSSQWQPLIQDRCFLSWLVKIPSEQEQLRARQITAQQINKLEELWK- 251
ENSPMEP00000030236_Pmex/1-1098  PCASQSSLKDINWDSSQWQPLIQDRCFLSWLVKIPSEQEQLRARQITAQQINKLEELWK- 251
ENSPLAP00000027526_Plat/1-1097  PCASQSSLKDINWDSSQWQPLIQDRCFLSWLVKIPSEQEQLRARQITAQQINKLEELWK- 251
ENSGAFP00000007158_Gaff/1-1097  PCASQSSLKDINWDSSQWQPLIQDRCFLSWLVKIPSEQEQLRARQITAQQINKLEELWK- 251
ENSBSLP00000000983_Bspl/1-1121  PCASQSSLKDINWDSSQWQPLIQDRCFLSWLVKIPSEQEQLRARQITAQQINKLEELWK- 266
ENSACIP00000014157_Acit/1-1024  PCASQSSLKDINWDSSQWQPLIQDRCFLSWLVKIPSEQEQLRARQITAQQINKLEELWK- 177
ENSSAUP00010022158_Saur/1-1106  PCASQSSLKDINWDSSQWQPLIQDRCFLSWLVKIPSEQEQLRARQITAQQINKLEELWK- 256
ENSSLUP00000031277_Sluc/1-1117  PCASQSSLKDINWDSSQWQPLIQDRCFLSWLVKIPSEQEQLRARQITAQQINKLEELWK- 269
ENSPRNP00000036031_Pran/1-1122  PCASQSSLKDINWDSSQWQPLIQDRCFLSWLVKIPSEQEQLRARQITAQQINKLEELWK- 273
ENSHCOP00000021761_Hcom/1-1100  PCASQSSLKDINWDSSQWQPLIQDRCFLSWLVKIPSEQEQLRARQITAQQINKLEELWK- 251
ENSGACP00000010750_Gacu/1-1114  PCASQSSLKDINWDSSQWQPLIQDRCFLSWLVKIPSEQEQLRARQITAQQINKLEELWK- 251
ENSCSEP00000025320_Csem/1-1098  PCASQSSLKDINWDSSQWQPLIQDRCFLSWLVKIPSEQEQLRARQITAQQINKLEELWK- 250
ENSCLMP00005001105_Clum/1-1122  PCASQSSLKDINWDSSQWQPLIQDRCFLSWLVKIPSEQEQLRARQITAQQINKLEELWK- 258
ENSENLP00000003285_Enau/1-1103  PCASQSSLKDINWDSSQWQPLIQDRCFLSWLVKIPSEQEQLRARQITAQQINKLEELWK- 252
ENSSORP00005051579_Sorb/1-1102  PCASQSSLKDINWDSSQWQPLIQDRCFLSWLVKIPSEQEQLRARQITAQQINKLEELWK- 251
ENSLBEP00000037352_Lber/1-1102  PCASQSSLKDINWDSSQWQPLIQDRCFLSWLVKIPSEQEQLRSRQITAQQINKLEELWK- 251
ENSONIP00000058998_Onil/1-1118  PCASQSSLKDINWDSSQWQPLIQDRCFLSWLVKIPSEQEQLRARQITAQQINKLEELWK- 263
ENSOABP00000030065_Oaur/1-1118  PCASQSSLKDINWDSSQWQPLIQDRCFLSWLVKIPSEQEQLRARQITAQQINKLEELWK- 263
ENSMMOP00000009727_Mmol/1-1024  PCASQSSLKDINWDSSQWQPLIQDRCFLSWLVKIPSEQEQLRARQITAQQINKLEELWK- 197
ENSCGOP00000030248_Cgob/1-1117  PCASQSSLKDINWDSSQWQPLIQDRCFLSWLVKIPSEQEQLRARQITAQQINKLEELWK- 267
ENSMZEP00005021871_Mzeb/1-1101  PCASQSSLKDINWDSSQWQPLIQDRCFLSWLVKIPSEQEQLRARQITAQQINKLEELWK- 251
ENSACLP00000033452_Acal/1-1101  PCASQSSLKDINWDSSQWQPLIQDRCFLSWLVKIPSEQEQLRARQITAQQINKLEELWK- 251
ENSNBRP00000029009_Nbri/1-1142  PCASQSSLKDINWDSSQWQPLIQDRCFLSWLVKIPSEQEQLRARQITAQQINKLEELWK- 251
ENSPNYP00000022183_Pnye/1-1101  PCASQSSLKDINWDSSQWQPLIQDRCFLSWLVKIPSEQEQLRARQITAQQINKLEELWK- 251
ENSSPAP00000015326_Spar/1-1100  PCASQSSLKDINWDSSQWQPLIQDRCFLSWLVKIPSEQEQLRARQITAQQINKLEELWK- 251
ENSSMAP00000034355_Smax/1-1102  PCASQSSLKDINWDSSQWQPLIQDRCFLSWLVKIPSEQEQLRARQITAQQINKLEELWK- 251
ENSMAMP00000009271_Marm/1-1102  PCASQSSLKDINWDSSQWQPLIQDRCFLSWLVKIPSEQEQLRARQITAQQINKLEELWK- 251
ENSLCRP00005054794_Lcro/1-1114  PCASQSSLKDINWDSSQWQPLIQDRCFLSWLVKIPSEQEQLRARQITAQQINKLEELWK- 251
ENSAOCP00000021516_Aoce/1-1101  PCASQSSLKDINWDSSQWQPLIQDRCFLSWLVKIPSEQEQLRARQITAQQINKLEELWK- 251
ENSAPEP00000008783_Aper/1-1101  PCASQSSLKDINWDSSQWQPLIQDRCFLSWLVKIPSEQEQLRARQITAQQINKLEELWK- 251
ENSATEP00000023978_Ates/1-1101  PCASQSSLKDINWDSSQWQPLIQDRCFLSWLVKIPSEQEQLRARQITAQQINKLEELWK- 251
ENSSLDP00000008708_Sldo/1-1102  PCASQSSLKDINWDSSQWQPLIQDRCFLSWLVKIPSEQEQLRARQITAQQINKLEELWK- 251
ENSSDUP00000013281_Sdum/1-1102  PCASQSSLKDINWDSSQWQPLIQDRCFLSWLVKIPSEQEQLRARQITAQQINKLEELWK- 251
ENSAPOP00000008674_Apol/1-1101  PCASQSSLKDINWDSSQWQPLIQDRCFLSWLVKIPSEQEQLRARQITAQQINKLEELWK- 251
ENSOTSP00005053122_Otsh/1-1117  PCASQSSLKDINWDSSQWQPLIQDRCFLSWLVKIPSEQEQLRARQITAQQINKLEELWK- 266
ENSOKIP00005034021_Okis/1-1117  PCASQSSLKDINWDSSQWQPLIQDRCFLSWLVKIPSEQEQLRARQITAQQINKLEELWK- 266
```

```
ENSSTUP00000016247_Stru/1-1117  PCASQSSLKDINWDSSQWQPLIQDRCFLSWLVKIPSEQEQLRARQITAQQINKLEELWK- 266
ENSHHUP00000024349_Hhuc/1-1107  PCASQSSLKDINWDSSQWQPLIQDRCFLSWLVKIPSEQEQLRARQITAQQINKLEELWK- 250
ENSSSAP00000049906_Ssal/1-1107  PCASQSSLKDINWDSSQWQPLIQDRCFLSWLVKIPSEQEQLRARQITAQQINKLEELWK- 250
ENSOMYP00000068529_Omyk/1-1107  PCASQSSLKDINWDSSQWQPLIQDRCFLSWLVKIPSEQEQLRARQITAQQINKLEELWK- 250
ENSELUP00000034330_Eluc/1-1110  PCASQSSLKDINWDSSQWQPLIQDRCFLSWLVKIPSEQEQLRARQITAQQINKLEELWK- 250
ENSSTUP00000015572_Stru/1-1119  PCASQSSLKDINWDSSQWQPLIQDRCFLSWLVKIPSEQEQLRARQITAQQINKLEELWK- 271
ENSHHUP00000047946_Hhuc/1-1116  PCASQSSLKDINWDSSQWQPLIQDRCFLSWLVKIPSEQEQLRARQITAQQINKLEELWK- 259
ENSSSAP00000095751_Ssal/1-1107  PCASQSSLKDINWDSSQWQPLIQDRCFLSWLVKIPSEQEQLRARQITAQQINKLEELWK- 250
ENSOMYP00000001570_Omyk/1-1107  PCASQSSLKDINWDSSQWQPLIQDRCFLSWLVKIPSEQEQLRARQITAQQINKLEELWK- 250
ENSOTSP00005066673_Otsh/1-1107  PCASQSSLKDINWDSSQWQPLIQDRCFLSWLVKIPSEQEQLRARQITAQQINKLEELWK- 250
ENSOKIP00005074715_Okis/1-1121  PCASQSSLKDINWDSSQWQPLIQDRCFLSWLVKIPSEQEQLRARQITAQQINKLEELWK- 267
ENSCHAP00000040423_Char/1-1113  PCASQSSLKDINWDSSQWQPLIQDRCFLSWLVKIPSEQEQLRARQIAAQQINKLEELWK- 251
ENSSFOP00015072781_Sfor/1-1139  PCASQSSLKDINWDSSQWQPLIQDRCFLSWLVKIPSEQEQLRARQITAQQINKLEELWK- 290
ENSSGRP00000083200_Sgra/1-1116  PCASQSSLKDINWDSSQWQPLIQDRCFLSWLVKIPSEQEQLRARQITAQQINKLEELWK- 257
ENSCARP00000022002_Caur/1-1108  PCASQSSLKDINWDSSQWQPLIQDRCFLSWLVKIPSEQEQLRARQITAQQINKLEELWK- 249
ENSCARP00000025658_Caur/1-1106  PCASQSSLKDINWDSSQWQPLIQDRCFLSWLVKIPSEQEQLRARQITAQQINKLEELWK- 249
ENSCCRP00010113915_Ccge/1-1102  PCASQSSLKDINWDSSQWQPLIQDRCFLSWLVKIPSEQEQLRARQITAQQINKLEELWK- 249
ENSCCRP00015080309_Cchu/1-1102  PCASQSSLKDINWDSSQWQPLIQDRCFLSWLVKIPSEQEQLRARQITAQQINKLEELWK- 249
ENSDARP00000153550_Drer/1-1100  PCASQSSLKDINWDSSQWQPLIQDRCFLSWLVKIPSEQEQLRARQITAQQINKLEELWK- 249
ENSSANP00000022280_Sans/1-1099  PCASQSSLKDINWDSSQWQPLIQDRCFLSWLVKIPSEQEQLRARQITAQQINKLEELWK- 249
ENSSRHP00000017387_Srhi/1-1099  PCASQSSLKDINWDSSQWQPLIQDRCFLSWLVKIPSEQEQLRARQITAQQINKLEELWK- 249
ENSSANP00000080941_Sans/1-1094  PCASQSSLKDINWDSSQWQPLIQDRCFLSWLVKIPSEQEQLRARQITAQQINKLEELWK- 249
ENSCCRP00010034499_Ccge/1-1100  PCASQSSLKDINWDSSQWQPLIQDRCFLSWLVKIPSEQEQLRARQITAQQINKLEELWK- 249
ENSCARP00000085195_Caur/1-1100  PCASQSSLKDINWDSSQWQPLIQDRCFLSWLVKIPSEQEQLRARQITAQQINKLEELWK- 249
ENSCARP00000002677_Caur/1-1100  PCASQSSLKDINWDSSQWQPLIQDRCFLSWLVKIPSEQEQLRARQITAQQINKLEELWK- 249
ENSCCRP00020094499_Cche/1-1100  PCASQSSLKDINWDSSQWQPLIQDRCFLSWLVKIPSEQEQLRARQITAQQINKLEELWK- 249
ENSCHAP00000025229_Char/1-1111  PCASQSSLKDINWDSSQWQPLIQDRCFLSWLVKIPSEQEQLRARQITAQQINKLEELWK- 261
ENSDCDP00000025137_Dclu/1-1099  PCASQSSLKDINWDSSQWQPLIQDRCFLSWLVKIPSEQEQLRARQITAQQINKLEELWK- 249
ENSEEEP00000000105_Eele/1-1097  PCASQSSLKDINWDSSQWQPLIQDRCFLSWLVKIPSEQEQLRARQITAQQINKLEELWK- 238
ENSIPUP00000007528_Ipun/1-1100  PCASQSSLKDINWDSSQWQPLIQDRCFLSWLVKIPSEQEQLRARQITAQQINKLEELWK- 249
ENSPNAP00000019041_Pnat/1-1100  PCASQSSLKDINWDSSQWQPLIQDRCFLSWLVKIPSEQEQLRARQITAQQINKLEELWK- 249
ENSAMXP00000051219_Amex/1-1100  PCASQSSLKDINWDSSQWQPLIQDRCFLSWLVKIPSEQEQLRARQITAQQINKLEELWK- 249
ENSFCAP00000058732_Fcat/1-1162  PCASQSSLKDINWDSSQWQPLIQDRCFLSWLVKIPSEQEQLRARQITAQQINKLEELWK- 271
ENSRFEP00010031518_Rfer/1-1169  PCASQSSLKDINWDSSQWQPLIQDRCFLSWLVKIPSEQEQLRARQITAQQINKLEELWK- 271
ENSOARP00000011525_Oari/1-1084  PCASQSSLKDINWDSSQWQPLIQDRCFLSWLVKIPSEQEQLRARQITAQQINKLEELWK- 264
ENSCCAP00000028528_Ccap/1-1151  PCASQSSLKDINWDSSQWQPLIQDRCFLSWLVKIPSEQEQLRARQITAQQINKLEELWK- 270
ENSDORP00000027782_Dord/1-1106  PCASQSSLKDINWDSSQWQPLIQDRCFLSWLVKIPSEQEQLRARQITAQQINKLEELWK- 254
ENSPPYP00000010923_Pabe/1-1076  PCASQSSLKDINWDSSQWQPLIQDRCFLSWLVKIPSEQEQLRARQITAQQINKLEELWK- 270
ENSCABP00000022072_Cabi/1-1095  PCASQSSLKDINWDSSQWQPLIQDRCFLSWLVKIPSEQEQLRARQITAQQINKLEELWK- 267
ENSPSTP00000010791_Pcri/1-1002  PCASQSSLKDINWDSSQWQPLIQDRCFLSWLVKIPSEQEQLRARQITAQQINKLEELWK- 141
ENSZCAP00015016162_Zcal/1-1102  PCASQSSLKDINWDSSQWQPLIQDRCFLSWLVKIPSEQEQLRARQITAQQINKLEELWK- 255
ENSHGLP00100007922_Hgma/1-1103  PCASQSSLKDINWDSSQWQPLIQDRCFLSWLVKIPSEQEQLRARQITAQQINKLEELWK- 219
ENSMUNP00000010143_Mund/1-1046  PCASQSSLKDINWDSSQWQPLIQDRCFLSWLVKIPSEQEQLRARQITAQQINKLEELWK- 224
ENSCMIP00000012225_Cmil/1-1067  PCASQSSLKDINWDSSQWQPLIQDRCFLSWLVKIPSEQEQLRARQITAQQINKLEELWK- 209
ENSCUSP00005010789_Cust/1-1090  PCASQSSLKDINWDSSQWQPLIQDRCFLSWLVKIPSEQEQLRARQITAQQINKLEELWK- 269
ENSCRFP00000000636_Sruf/1-1122  PCASQSSLKDINWDSSQWQPLIQDRCFLSWLVKIPSEQEQLRARQITAQQINKLEELWK- 269
ENSTTRP00000010948_Ttru/1-1112  PCASQSSLKDINWSS-QWQ-LIQDRCFLSWLVI--SEQEQLRARQITAQQINKLEELCK- 264
ENSBMUP00000020134_Bmut/1-1130  PCASQSSLKDINWDSSQWQPLIQDRCFLSWLVKIPSEQEQLRARQITAQQINKLEELWK- 271
ENSANAP00000012377_Anan/1-1092  PCASQSSLKDINWDSSQWQPLIQDRCFLSWLVKIPSEQEQLRARQITAQQINKLEELWK- 247
ENSMAUP00000010607_Maur/1-1108  PCASQSSLKDINWDSSQWQPLIQDRCFLSWLVKIPSEQEQLRARQITAQQINKLEELWK- 249
ENSPEMP00000001379_Pmba/1-1124  PCASQSSLKDINWDSSQWQPLIQDRCFLSWLVKIPSEQEQLRARQITAQQINKLEELWK- 265
ENSCAPP00000002851_Cape/1-1102  PCASQSSLKDINWDSSQWQPLIQDRCFLSWLVKIPSEQEQLRARQITAQQINKLEELWK- 243
ENSRBIP00000003024_Rbie/1-1068  PCASQSSLKDINWDSSQWQPLIQDRCFLSWLVKIPSEQEQLRARQITAQQINKLEELWK- 209
ENSDNVP00000017438_Dnov/1-1055  PCASQSSLKDINWDSSQWQPLIQDRCFLSWLVKIPSEQEQLRARQITAQQINKLEELWK- 229
ENSUMAP00000009637_Umar/1-1095  PCASQSSLKDINWDSSQWQPLIQDRCFLSWLVKIPSEQEQLRARQITAQQINKLEELWK- 247
ENSFDAP00000005923_Fdam/1-1096  PCASQSSLKDINWDSSQWQPLIQDRCFLSWLVKIPSEQEQLRARQITAQQINKLEELWK- 237
ENSPPAP00000042672_Ppan/1-1096  PCASQSSLKDINWDSSQWQPLIQDRCFLSWLVKIPSEQEQLRARQITAQQINKLEELWK- 237
ENSSBOP00000013846_Sbbo/1-1092  PCASQSSLKDINWDSSQWQPLIQDRCFLSWLVKIPSEQEQLRARQITAQQINKLEELWK- 233
ENSCLAP00000005692_Clan/1-1126  PCASQSSLKDINWDSSQWQPLIQDRCFLSWLVKIPSEQEQLRARQITAQQINKLEELWK- 267
ENSSSUP00005009320_Ssur/1-1110  PCASQSSLKDINWDSSQWQPLIQDRCFLSWLVKIPSEQEQLRARQITAQQINKLEELWK- 251
ENSBBBP00000021865_Bbbi/1-1130  PCASQSSLKDINWDSSQWQPLIQDRCFLSWLVKIPSEQEQLRARQITAQQINKLEELWK- 271
ENSTSYP00000017119_Csyr/1-1129  PCASQSSLKDINWDSSQWQPLIQDRCFLSWLVKIPSEQEQLRARQITAQQINKLEELWK- 270
ENSMVIP00005027631_Mvit/1-1099  PCASQSSLKDINWDSSQWQPLIQDRCFLSWLVKIPSEQEQLRARQITAQQINKLEELWK- 243
ENSACUP00000017471_Acun/1-1041  PCASQSSLKDINWDSSQWQPLIQDRCFLSWLVKIPSEQEQLRARQITAQQINKLEELWK- 202
ENSLCOP00000003495_Lcor/1-1113  PCASQSSLKDINWDSSQWQPLIQDRCFLSWLVKIPSEQEQLRARQITAQQINKLEELWK- 260
ENSFALP00000009727_Falb/1-1088  PCASQSSLKDINWDSSQWQPLIQDRCFLSWLVKIPSEQEQLRARQITAQQINKLEELWK- 239
ENSSCUP00000013837_Scau/1-1092  PCASQSSLKDINWDSSQWQPLIQDRCFLSWLGKIPSEQEQLRARQITAQQINKLEELWK- 232
ENSZLMP00000005403_Zlme/1-897   PCASQSSLKDINWDSSQWQPLIQDRCFLSWLVKIPSEQEQLRARQITAQQINKLEELWK- 101
ENSMCSP00000018063_Mcsa/1-1080  PCASQSSLKDINWDSSQWQPLIQDRCFLSWLVKIPSEQEQLRARQITAQQINKLEELWK- 222
ENSCGRP00000018518_Cgcr/1-1070  PCASQSSLKDINWDSSQWQPLIQDRCFLSWLVKIPSEQEQLRARQITAQQINKLEELWK- 211
ENSGGOP00000002572_Ggor/1-1054  PCASQSSLKDINWDSSQWQPLIQDRCFLSWLVKIPSEQEQLRARQITAQQINKLEELWK- 206
ENSPTIP00000008974_Ptal/1-1022  PCASQSSLKDINWDSSQWQPLIQDRCFLSWLVKIPSEQEQLRARQITAQQINKLEELWK- 195
```

```
ENSCATP00000034826_Caty/1-1073  PCASQSSLKDINWDSSQWQPLIQDRCFLSWLVKIPSEQEQLRARQITAQQINKLEELWK- 214
ENSSDAP00000011933_Sdau/1-1076  PCASQSSLKDINWDSSQWQPLIQDRCFLSWLVKIPSEQEQLRARQITAQQINKLEELWK- 217
ENSRROP00000034798_Rrox/1-1079  PCASQSSLKDINWDSSQWQPLIQDRCFLSWLVKIPSEQEQLRARQITAQQINKLEELWK- 220
ENSCANP00000013001_Capa/1-1079  PCASQSSLKDINWDSSQWQPLIQDRCFLSWLVKIPSEQEQLRARQITAQQINKLEELWK- 220
ENSMLEP00000008475_Mleu/1-1052  PCASQSSLKDINWDSSQWQPLIQDRCFLSWLVKIPSEQEQLRARQITAQQINKLEELWK- 193
ENSMGAP00000004600_Mgal/1-1052  PCASQSSLKDINWDSSQWQPLIQDRCFLSWLVKIPSEQEQLRARQITAQQINKLEELWK- 192
ENSSCAP00000016083_Scan/1-1066  PCASQSSLKDINWDSSQWQPLIQDRCFLSWLVKIPSEQEQLRARQITAQQINKLEELWK- 218
ENSCCEP00000002811_Ccae/1-1071  PCASQSSLKDINWDSSQWQPLIQDRCFLSWLVKIPSEQEQLRARQITAQQINKLEELWK- 219
ENSGFOP00000006283_Gfor/1-1051  PCASQSSLKDINWDSSQWQPLIQDRCFLSWLVKIPSEQEQLRARQITAQQINKLEELWK- 192
ENSSHAP00000012247_Shar/1-1053  PCASQSSLKDINWDSSQWQPLIQDRCFLSWLVKIPSEQEQLRARQITAQQINKLEELWK- 192
ENSPCIP00000007258_Pcin/1-1051  PCASQSSLKDINWDSSQWQPLIQDRCFLSWLVKIPSEQEQLRARQITAQQINKLEELWK- 192
ENSMODP00000040291_Mdom/1-1053  PCASQSSLKDINWDSSQWQPLIQDRCFLSWLVKIPSEQEQLRARQITAQQINKLEELWK- 194
ENSBJAP00000009539_Bjap/1-1058  PCASQSSLKDINWDSSQWQPLIQDRCFLSWLVKIPSEQEQLRARQITAQQINKLEELWK- 203
ENSAHAP00000016790_Ahaa/1-1080  PCASQSSLKDINWDSSQWQPLIQDRCFLSWLVKIPSEQEQLRARQITAQQINKLEELWK- 229
ENSAOWP00000005794_Aowe/1-1080  PCASQSSLKDINWDSSQWQPLIQDRCFLSWLVKIPSEQEQLRARQITAQQINKLEELWK- 229
ENSARWP00000022924_Arow/1-1080  PCASQSSLKDINWDSSQWQPLIQDRCFLSWLVKIPSEQEQLRARQITAQQINKLEELWK- 229
ENSCPUP00000022997_Cpug/1-942   PCASQSSLKDINWDSSQWQPLIQDRCFLSWLVKIPSEQEQLRARQITAQQINKLEELWK- 87
ENSBOBP00000014391_Bbub/1-1055  PCASQSSLKDINWDSSQWQPLIQDRCFLSWLVKIPSEQEQLRARQITAQQINKLEELWK- 197
ENSABRP00000005609_Abra/1-1059  PCASQSSLKDINWDSSQWQPLIQDRCFLSWLVKIPSEQEQLRARQITAQQINKLEELWK- 204
ENSSOCP00000002229_Soca/1-1042  PCASQSSLKDINWDSSQWQPLIQDRCFLSWLVKIPSEQEQLRARQITAQQINKLEELWK- 194
ENSAZOP00000006180_Azon/1-1042  PCASQSSLKDINWDSSQWQPLIQDRCFLSWLVKIPSEQEQLRARQITAQQINKLEELWK- 194
ENSOSUP00000016421_Osun/1-1046  PCASQSSLKDINWDSSQWQPLIQDRCFLSWLVKIPSEQEQLRARQITAQQINKLEELWK- 198
ENSVKKP00000018558_Vkom/1-1126  PCASQSSLKDINWDSSQWQPLIQDRCFLSWLVKIPSEQEQLRARQITAQQINKLEELWK- 269
ENSOANP00000007238_Oana/1-1113  PCASQSSLKDINWDSSQWQPLIQDRCFLSWLVKIPSEQEQLRARQITAQQINKLEELWK- 267
ENSSMRP00000029871_Smer/1-1319  PCASQSSLKDINWDSSQWQPLIQDRCFLSWLVKIPSEQEQLRARQITAQQINKLEELWK- 472
ENSPVIP00000019661_Pvit/1-1125  PCASQSSLKDINWDSSQWQPLIQDRCFLSWLVKIPSEQEQLRARQITAQQINKLEELWK- 268
ENSNSUP00000007525_Nscu/1-1137  PCASQSSLKDINWDSSQWQPLIQDRCFLSWLVKIPSEQEQLRARQITAQQINKLEELWK- 275
ENSNNAP00000002815_Nnaj/1-1131  PCASQSSLKDINWDSSQWQPLIQDRCFLSWLVKIPSEQEQLRARQITAQQINKLEELWK- 284
ENSPTXP00000003133_Ptex/1-1131  PCASQSSLKDINWDSSQWQPLIQDRCFLSWLVKIPSEQEQLRARQITAQQINKLEELWK- 284
ENSNPEP00000004095_Nper/1-1128  PCASQSSLKDINWDSSQWQPLIQDRCFLSWLVKIPSEQEQLRARQITAQQINKLEELWK- 280
ENSPMRP00000033661_Pmur/1-1131  PCASQSSLKDINWDSSQWQPLIQDRCFLSWLVKIPSEQEQLRARQITAQQINKLEELWK- 284
ENSSPUP00000013678_Spun/1-1109  PCASQSSLKDINWDSSQWQPLIQDRCFLSWLVKIPSEQEQLRARQITAQQINKLEELWK- 262
ENSACOP00000002466_Acol/1-1128  PCASQSSLKDINWDSSQWQPLIQDRCFLSWLVKIPSEQEQLRARQITAQQINKLEELWK- 270
ENSCPBP00000017033_Cpbe/1-1113  PCASQSSLKDINWDSSQWQPLIQDRCFLSWLVKIPSEQEQLRARQITAQQINKLEELWK- 262
ENSCPRP00005022544_Cpor/1-1117  PCASQSSLKDINWDSSQWQPLIQDRCFLSWLVKIPSEQEQLRARQITAQQINKLEELWK- 269
ENSCPVP00000019292_Cpar/1-1117  PCASQSSLKDINWDSSQWQPLIQDRCFLSWLVKIPSEQEQLRARQITAQQINKLEELWK- 269
ENSJHYP00000007239_Jhye/1-1117  PCASQSSLKDINWDSSQWQPLIQDRCFLSWLVKIPSEQEQLRARQITAQQINKLEELWK- 269
ENSTGUP00000001084_Tgut/1-1117  PCASQSSLKDINWDSSQWQPLIQDRCFLSWLVKIPSEQEQLRARQITAQQINKLEELWK- 269
ENSCMUP00000018250_Cmon/1-1117  PCASQSSLKDINWDSSQWQPLIQDRCFLSWLVKIPSEQEQLRARQITAQQINKLEELWK- 269
ENSCJPP00005013449_Cjap/1-1117  PCASQSSLKDINWDSSQWQPLIQDRCFLSWLVKIPSEQEQLRARQITAQQINKLEELWK- 269
ENSGALP00000005090_Ggal/1-1117  PCASQSSLKDINWDSSQWQPLIQDRCFLSWLVKIPSEQEQLRARQITAQQINKLEELWK- 269
ENSNMEP00000003287_Nmel/1-1117  PCASQSSLKDINWDSSQWQPLIQDRCFLSWLVKIPSEQEQLRARQITAQQINKLEELWK- 269
ENSPCLP00000015045_Pcol/1-1117  PCASQSSLKDINWDSSQWQPLIQDRCFLSWLVKIPSEQEQLRARQITAQQINKLEELWK- 269
ENSCPGP00000009322_Cpyg/1-1120  PCASQSSLKDINWDSSQWQPLIQDRCFLSWLVKIPSEQEQLRARQITAQQINKLEELWK- 269
ENSACDP00005024899_Acyg/1-1117  PCASQSSLKDINWDSSQWQPLIQDRCFLSWLVKIPSEQEQLRARQITAQQINKLEELWK- 269
ENSAPLP00000008896_Appl/1-1117  PCASQSSLKDINWDSSQWQPLIQDRCFLSWLVKIPSEQEQLRARQITAQQINKLEELWK- 269
ENSCMMP00000009287_Cmdo/1-1117  PCASQSSLKDINWDSSQWQPLIQDRCFLSWLVKIPSEQEQLRARQITAQQINKLEELWK- 269
ENSSHBP00005020370_Shab/1-1124  PCASQSSLKDINWDSSQWQPLIQDRCFLSWLVKIPSEQEQLRARQITAQQINKLEELWK- 269
ENSACCP00020008733_Acch/1-1117  PCASQSSLKDINWDSSQWQPLIQDRCFLSWLVKIPSEQEQLRARQITAQQINKLEELWK- 269
ENSFTIP00000010528_Ftin/1-1117  PCASQSSLKDINWDSSQWQPLIQDRCFLSWLVKIPSEQEQLRARQITAQQINKLEELWK- 269
ENSPCEP00000010643_Pcas/1-1113  PCASQSSLKDINWDSSQWQPLIQDRCFLSWLVKIPSEQEQLRARQITAQQINKLEELWK- 265
ENSGAGP00000030895_Gaga/1-1108  PCASQSSLKDINWDSSQWQPLIQDRCFLSWLVKIPSEQEQLRARQITAQQINKLEELWK- 260
ENSGEVP00005027471_Gevg/1-1108  PCASQSSLKDINWDSSQWQPLIQDRCFLSWLVKIPSEQEQLRARQITAQQINKLEELWK- 260
ENSTMTP00000017273_Tctr/1-1115  PCASQSSLKDINWDSSQWQPLIQDRCFLSWLVKIPSEQEQLRARQITAQQINKLEELWK- 267
ENSCSRP00000016962_Cser/1-1113  PCASQSSLKDINWDSSQWQPLIQDRCFLSWLVKIPSEQEQLRARQITAQQINKLEELWK- 265
ENSXETP00000068717_Xtro/1-1126  PCASQSSLKDINWDSSQWQPLIQDRCFLSWLVKIPSEQEQLRARQITAQQINKLEELWK- 277
ENSLLEP00000027898_Llei/1-1100  PCASQSSLKDINWDSSQWQPLIQDRCFLSWLVKIPSEQEQLRARQITAQQINKLEELWK- 251
ENSLOCP00000002426_Locu/1-1100  PCASQSSLKDINWDSSQWQPLIQDRCFLSWLVKIPSEQEQLRARQITAQQINKLEELWK- 251
ENSECRP00000018059_Ecal/1-1100  PCASQSSLKDINWDSSQWQPLIQDRCFLSWLVKIPSEQEQLRARQITAQQINKLEELWK- 251
ENSLAFP00000023305_Lafr/1-1129  PCASQSSLKDINWDSSQWQPLIQDRCFLSWLVKIPSEQEQLRARQITAQQINKLEELWK- 270
ENSMICP00000001513_Mmur/1-1119  PCASQSSLKDINWDSSQWQPLIQDRCFLSWLVKIPSEQEQLRARQITAQQINKLEELWK- 270
ENSCPOP00000015668_Cpor/1-1109  PCASQSSLKDINWDSSQWQPLIQDRCFLSWLVKIPSEQEQLRARQITAQQINKLEELWK- 250
ENSJJAP00000023360_Jjac/1-1122  PCASQSSLKDINWDSSQWQPLIQDRCFLSWLVKIPSEQEQLRARQITAQQINKLEELWK- 263
ENSNGAP00000026472_Ngal/1-1127  PCASQSSLKDINWDSSQWQPLIQDRCFLSWLVKIPSEQEQLRARQITAQQINKLEELWK- 268
ENSMUGP00000015658_Mung/1-1113  PCASQSSLKDINWDSSQWQPLIQDRCFLSWLVKIPSEQEQLRARQITAQQINKLEELWK- 265
ENSMOCP00000006123_Moch/1-1124  PCASQSSLKDINWDSSQWQPLIQDRCFLSWLVKIPSEQEQLRARQITAQQINKLEELWK- 265
ENSCGRP00001022573_Cgch/1-1113  PCASQSSLKDINWDSSQWQPLIQDRCFLSWLVKIPSEQEQLRARQITAQQINKLEELWK- 265
ENSCGRP00015000072_Cgpi/1-1113  PCASQSSLKDINWDSSQWQPLIQDRCFLSWLVKIPSEQEQLRARQITAQQINKLEELWK- 265
ENSRNOP00000075905_Rnor/1-1124  PCASQSSLKDINWDSSQWQPLIQDRCFLSWLVKIPSEQEQLRARQITAQQINKLEELWK- 265
ENSMSIP00000038476_Mspi/1-1124  PCASQSSLKDINWDSSQWQPLIQDRCFLSWLVKIPSEQEQLRARQITAQQINKLEELWK- 265
ENSMUSP00000075089_Mmus/1-1124  PCASQSSLKDINWDSSQWQPLIQDRCFLSWLVKIPSEQEQLRARQITAQQINKLEELWK- 265
ENSCCNP00000004597_Ccan/1-1129  PCASQSSLKDINWDSSQWQPLIQDRCFLSWLVKIPSEQEQLRARQITAQQINKLEELWK- 270
```

```
ENSVURP00010019905_Vurs/1-1129  PCASQSSLKDINWDSSQWQPLIQDRCFLSWLVKIPSEQEQLRARQITAQQINKLEELWK- 270
ENSCHIP00000007632_Chir/1-1126  PCASQSSLKDINWDSSQWQPLIQDRCFLSWLVKIPSEQEQLRARQITAQQINKLEELWK- 267
ENSDLEP00000008365_Dleu/1-1127  PCASQSSLKDINWDSSQWQPLIQDRCFLSWLVKIPSEQEQLRARQITAQQINKLEELWK- 268
ENSMMNP00015009574_Mmon/1-1127  PCASQSSLKDINWDSSQWQPLIQDRCFLSWLVKIPSEQEQLRARQITAQQINKLEELWK- 268
ENSBMSP00010005595_Bmus/1-1127  PCASQSSLKDINWDSSQWQPLIQDRCFLSWLVKIPSEQEQLRARQITAQQINKLEELWK- 268
ENSPCTP00005027959_Pcat/1-1127  PCASQSSLKDINWDSSQWQPLIQDRCFLSWLVKIPSEQEQLRARQITAQQINKLEELWK- 268
ENSPSNP00000002932_Psin/1-1127  PCASQSSLKDINWDSSQWQPLIQDRCFLSWLVKIPSEQEQLRARQITAQQINKLEELWK- 268
ENSSSCP00000036535_Sscr/1-1127  PCASQSSLKDINWDSSQWQPLIQDRCFLSWLVKIPSEQEQLRARQITAQQINKLEELWK- 268
ENSCDRP00005012630_Cdro/1-1127  PCASQSSLKDINWDSSQWQPLIQDRCFLSWLVKIPSEQEQLRARQITAQQINKLEELWK- 268
ENSBTAP00000027868_Btau/1-1127  PCASQSSLKDINWDSSQWQPLIQDRCFLSWLVKIPSEQEQLRARQITAQQINKLEELWK- 268
ENSBIXP00000023236_Bthy/1-1127  PCASQSSLKDINWDSSQWQPLIQDRCFLSWLVKIPSEQEQLRARQITAQQINKLEELWK- 268
ENSBIXP00005007580_Bihy/1-1127  PCASQSSLKDINWDSSQWQPLIQDRCFLSWLVKIPSEQEQLRARQITAQQINKLEELWK- 268
ENSBGRP00000001520_Bgru/1-1127  PCASQSSLKDINWDSSQWQPLIQDRCFLSWLVKIPSEQEQLRARQITAQQINKLEELWK- 268
ENSCHYP00000023500_Chya/1-1127  PCASQSSLKDINWDSSQWQPLIQDRCFLSWLVKIPSEQEQLRARQITAQQINKLEELWK- 268
ENSCWAP00000025669_Cwag/1-1127  PCASQSSLKDINWDSSQWQPLIQDRCFLSWLVKIPSEQEQLRARQITAQQINKLEELWK- 268
ENSMMSP00000030114_Mmos/1-1127  PCASQSSLKDINWDSSQWQPLIQDRCFLSWLVKIPSEQEQLRARQITAQQINKLEELWK- 268
ENSHGLP00000015239_Hgfe/1-1170  PCASQSSLKDINWDSSQWQPLIQDRCFLSWLVKIPSEQEQLRARQITAQQINKLEELWK- 270
ENSAMEP00000012088_Amel/1-1125  PCASQSSLKDINWDSSQWQPLIQDRCFLSWLVKIPSEQEQLRARQITAQQINKLEELWK- 266
ENSODEP00000016858_Odeg/1-1110  PCASQSSLKDINWDSSQWQPLIQDRCFLSWLVKIPSEQEQLRARQITAQQINKLEELWK- 251
ENSMLUP00000011055_Mluc/1-1118  PCASQSSLKDINWDSSQWQPLIQDRCFLSWLVKIPSEQEQLRARQITAQQINKLEELWK- 270
ENSSVLP00005020593_Svul/1-1129  PCASQSSLKDINWDSSQWQPLIQDRCFLSWLVKIPSEQEQLRARQITAQQINKLEELWK- 270
ENSSTOP00000030217_Itri/1-1152  PCASQSSLKDINWDSSQWQPLIQDRCFLSWLVKIPSEQEQLRARQITAQQINKLEELWK- 270
ENSUPAP00010023106_Upar/1-1118  PCASQSSLKDINWDSSQWQPLIQDRCFLSWLVKIPSEQEQLRARQITAQQINKLEELWK- 270
ENSPSMP00000001497_Psim/1-1129  PCASQSSLKDINWDSSQWQPLIQDRCFLSWLVKIPSEQEQLRARQITAQQINKLEELWK- 270
ENSOGAP00000011534_Ogar/1-1118  PCASQSSLKDINWDSSQWQPLIQDRCFLSWLVKIPSEQEQLRARQITAQQINKLEELWK- 270
ENSCSAP00000001590_Csab/1-1118  PCASQSSLKDINWDSSQWQPLIQDRCFLSWLVKIPSEQEQLRARQITAQQINKLEELWK- 270
ENSMNEP00000000047_Mnem/1-1129  PCASQSSLKDINWDSSQWQPLIQDRCFLSWLVKIPSEQEQLRARQITAQQINKLEELWK- 270
ENSTGEP00000018597_Tgel/1-1129  PCASQSSLKDINWDSSQWQPLIQDRCFLSWLVKIPSEQEQLRARQITAQQINKLEELWK- 270
ENSPTRP00000069011_Ptro/1-1129  PCASQSSLKDINWDSSQWQPLIQDRCFLSWLVKIPSEQEQLRARQITAQQINKLEELWK- 270
ENSMFAP00000037204_Mfas/1-1129  PCASQSSLKDINWDSSQWQPLIQDRCFLSWLVKIPSEQEQLRARQITAQQINKLEELWK- 270
ENSPANP00000032997_Panu/1-1129  PCASQSSLKDINWDSSQWQPLIQDRCFLSWLVKIPSEQEQLRARQITAQQINKLEELWK- 270
ENSPTEP00000029996_Ptep/1-1118  PCASQSSLKDINWDSSQWQPLIQDRCFLSWLVKIPSEQEQLRARQITAQQINKLEELWK- 270
ENSP00000470142_Hsap/1-1129     PCASQSSLKDINWDSSQWQPLIQDRCFLSWLVKIPSEQEQLRARQITAQQINKLEELWK- 270
ENSCJAP00000036494_Cjac/1-1129  PCASQSSLKDINWDSSQWQPLIQDRCFLSWLVKIPSEQEQLRARQITAQQINKLEELWK- 270
ENSMMUP00000043788_Mmul/1-1129  PCASQSSLKDINWDSSQWQPLIQDRCFLSWLVKIPSEQEQLRARQITAQQINKLEELWK- 270
ENSPCOP00000023360_Pcoq/1-1129  PCASQSSLKDINWDSSQWQPLIQDRCFLSWLVKIPSEQEQLRARQITAQQINKLEELWK- 270
ENSEASP00005009145_Eaas/1-1116  PCASQSSLKDINWDSSQWQPLIQDRCFLSWLVKIPSEQEQLRARQITAQQINKLEELWK- 268
ENSECAP00000008885_Ecab/1-1127  PCASQSSLKDINWDSSQWQPLIQDRCFLSWLVKIPSEQEQLRARQITAQQINKLEELWK- 268
ENSUAMP00000008400_Uame/1-1119  PCASQSSLKDINWDSSQWQPLIQDRCFLSWLVKIPSEQEQLRARQITAQQINKLEELWK- 271
ENSUTTP00000012254_Utth/1-1130  PCASQSSLKDINWDSSQWQPLIQDRCFLSWLVKIPSEQEQLRARQITAQQINKLEELWK- 271
ENSMPUP00000015404_Mpfu/1-1119  PCASQSSLKDINWDSSQWQPLIQDRCFLSWLVKIPSEQEQLRARQITAQQINKLEELWK- 271
ENSVVUP00000010553_Vvul/1-1119  PCASQSSLKDINWDSSQWQPLIQDRCFLSWLVKIPSEQEQLRARQITAQQINKLEELWK- 271
ENSNVIP00000025712_Nvis/1-1119  PCASQSSLKDINWDSSQWQPLIQDRCFLSWLVKIPSEQEQLRARQITAQQINKLEELWK- 271
ENSCAFP00020015362_Cldi/1-1119  PCASQSSLKDINWDSSQWQPLIQDRCFLSWLVKIPSEQEQLRARQITAQQINKLEELWK- 271
ENSCAFP00000021524_Clfa/1-1130  PCASQSSLKDINWDSSQWQPLIQDRCFLSWLVKIPSEQEQLRARQITAQQINKLEELWK- 271
ENSCAFP00040036533_Clfa/1-1130  PCASQSSLKDINWDSSQWQPLIQDRCFLSWLVKIPSEQEQLRARQITAQQINKLEELWK- 271
ENSCAFP00030024483_Clfa/1-1130  PCASQSSLKDINWDSSQWQPLIQDRCFLSWLVKIPSEQEQLRARQITAQQINKLEELWK- 271
ENSPPRP00000002368_Ppar/1-1130  PCASQSSLKDINWDSSQWQPLIQDRCFLSWLVKIPSEQEQLRARQITAQQINKLEELWK- 271
ENSLCNP00005010249_Lcan/1-1130  PCASQSSLKDINWDSSQWQPLIQDRCFLSWLVKIPSEQEQLRARQITAQQINKLEELWK- 271
ENSPLOP00000000736_Pleo/1-1130  PCASQSSLKDINWDSSQWQPLIQDRCFLSWLVKIPSEQEQLRARQITAQQINKLEELWK- 271
```

Supplementary Table S2. PCR primers utilized in the study.

Gene	Primer	Sequence
c-myc	F-primer<br>R-primer	AGCAGCGACTCTGAGGAGGAAC<br>TCCAGCAGAAGGTGATCCAGACT
p53	F-primer<br>R-primer	GCTCAGATAGCGATGGTCTGGC<br>CTCATAGGGCACCACCACACT
notch-1	F-primer<br>R-primer	GCATCTGTGCCAGTACGATGTGG<br>CCGTGTACCCTTCCGTGCA
hey-1	F-primer<br>R-primer	CATACGGCAGGAGGGAAAGGTTAC<br>AAGCGGGTCAGAGGCATCTAGT
hey-2	F-primer<br>R-primer	GCAACAGGGGGTAAAGGCTACT<br>AGATGAGACACAAGCCGCACC
hes-1	F-primer<br>R-primer	GGATGCTCTGAAGAAAGATAGCTCGC<br>CGGAGGTGCTTCACTGTCATTTCC
nAS25	RT primer<br>F-primer<br>R-primer	CTCTTGTGCCACATCCTGGACTACAG<br>CAGGGGTCATTGAAGTTGAGGGAG<br>CTCTTGTGCCACATCCTGGACTACAG
Exon25-notch-1	F-primer<br>R-primer	CTCTTGTGCCACATCCTGGACTACAG<br>CAGGGGTCATTGAAGTTGAGGGAG
Exon5/6-notch-1	F-primer	CGCCAGAGTGGACAGGTCAGTA
Exon 33-notch-1	R-primer	CATATCTTTGTTAGCCCCGTTCTTCAGGAG
Apobec1	F-primer<br>R-primer	GAGCATCAGAGTATTATCACTGCTGGAGGAA<br>ACAGGGTGGAAGACTTAGAATTATGAAGTGC
β-actin	F-primer<br>R-primer	AGAGCTACGAGCTGCCTGACG<br>GGACTCCATGCCCAGGAAGGA
